# Interaction Analysis of Longevity Interventions Using Survival Curves

**DOI:** 10.3390/biology7010006

**Published:** 2018-01-06

**Authors:** Stefan Nowak, Johannes Neidhart, Ivan G. Szendro, Jonas Rzezonka, Rahul Marathe, Joachim Krug

**Affiliations:** 1 Systems Biology of Ageing Cologne (Sybacol), University of Cologne, 50931 Cologne, Germany; sn@sqix.de (S.N.); jneidhart@zoho.com (J.N.); igst@yahoo.com (I.G.S.); Jonas.Rzez@web.de (J.R.); maratherahul@physics.iitd.ac.in (R.M.); 2 Institut für Theoretische Physik, Universität zu Köln, 50937 Cologne, Germany; 3 MBR Optical Systems, 42279 Wuppertal, Germany; 4 Department of Physics, Indian Institute of Technology Delhi, Hauz Khas, 110016 New Delhi, India

**Keywords:** models of ageing, longevity interventions, epistasis, survival curves, failure time analysis, *Caenorhabditis elegans*

## Abstract

A long-standing problem in ageing research is to understand how different factors contributing to longevity should be expected to act in combination under the assumption that they are independent. Standard interaction analysis compares the extension of mean lifespan achieved by a combination of interventions to the prediction under an additive or multiplicative null model, but neither model is fundamentally justified. Moreover, the target of longevity interventions is not mean life span but the entire survival curve. Here we formulate a mathematical approach for predicting the survival curve resulting from a combination of two independent interventions based on the survival curves of the individual treatments, and quantify interaction between interventions as the deviation from this prediction. We test the method on a published data set comprising survival curves for all combinations of four different longevity interventions in *Caenorhabditis elegans*. We find that interactions are generally weak even when the standard analysis indicates otherwise.

## 1. Introduction

Research on the biology of ageing has revealed a large variety of genetic, metabolic and environmental interventions that increase lifespan in model organisms [1,2,3,4,5]. Some interventions, such as dietary restriction, are remarkably universal and apply in similar form across widely different species [6,7]. An important tool that is used to unravel the underlying mechanisms is epistasis analysis, where the effect of a given intervention on lifespan is probed in the presence of another manipulation [7,8,9,10]. In molecular and population genetics the term epistasis commonly refers to interactions between the effects of genetic mutations [11,12,13,14,15], but here we will consider a broader range of effects that includes also physiological interventions. The interpretation of epistasis studies is relatively straightforward if the effect of the first intervention either persists unchanged or is completely masked by the second, where the latter outcome corresponds to the original meaning of the word epistasis [12]. However, in many cases the mutual influence of different interventions is quantitative rather than qualitative, and correspondingly a quantitative criterion of independence is required in order to infer whether and how the interventions interact. In the following, we will use the term *interaction* to emphasize our focus on such quantitative changes, and to delimit our approach from the traditional understanding of epistasis as the complete inhibition of the effects of one intervention by another.

In the past, most interaction studies have focused on mean or median lifespan as the primary longevity phenotype. These studies typically employ a plausible null model [16] where either the absolute lifespan extensions caused by independent interventions are assumed to add up (*additive model*), or the relative increases are assumed to multiply (*multiplicative model*). No clear preference for either of the two null models can be derived from first principles. It has therefore been recommended that both the additive and multiplicative scales should be used to test for interactions in longevity data [9]. More importantly, the restriction to mean lifespan for the quantification of longevity effects neglects the entire information contained in the shape of the survival curve [17,18,19]. Many studies have incorporated shape information by fitting experimental survival curves to mathematical models [20,21,22,23,24,25,26,27]. However, this approach has only rarely been used to analyze interactions in terms of model parameters such as the rate of mortality acceleration [10]. A framework for interaction analysis that is based directly on the survival curve does not seem to have been proposed previously.

For the following discussion, a survival curve S(x) is a monotonically decreasing function that quantifies the fraction of the population that is still alive at time *x*. Accordingly, S(x) is restricted to the interval [0,1] with limits S(0)=1 and S(x→∞)=0. Then, the purpose of this paper is to address the following question: Given a baseline survival curve $S_{0}\left(x\right)$ and survival curves $S_{1}\left(x\right)$ and $S_{2}\left(x\right)$ resulting from two different longevity interventions, can one predict the survival curve $S_{12}\left(x\right)$ that would result if the two interventions acted in combination and independently? We propose several possible answers to this question that are based on different assumptions about the meaning of independence, and which we collectively refer to as *composition principles* (*CPs*).

Adopting the view that epistatic interactions, in the most general sense of the term, express “our surprise at the phenotype when mutations are combined, given the constituent mutations’ individual effects” [15], the validity of a CP implies the absence of interactions on the level of the survival curves. Correspondingly, the deviation of the data from the prediction of the CPs can be used to quantify the amount of interactions. The implementation of this idea requires us to formalize the effect of a given longevity intervention as a mathematical transformation acting on the set of survival curves. As a simple example, consider the temporal rescaling operation S(x)→S(bx), where b<1 if lifespan is increased [28]. If $S_{1}\left(x\right)$ and $S_{2}\left(x\right)$ arise from the baseline survival curve $S_{0}\left(x\right)$ by temporal rescaling with factors $b_{1}$ and $b_{2}$, respectively, then the natural prediction for the survival curve of the combined intervention, under the assumption that the two interventions do not interact, is obtained by *composing* the two rescaling operations as

(1) $$S_{12}\left(x\right)=S_{1}\left(b_{2}x\right)=S_{2}\left(b_{1}x\right)=S_{0}\left(b_{1}b_{2}x\right).$$

Note that the empirical validity of this relation is far from obvious, even if all four survival curves are indeed related by temporal scaling. In practice, we have found that simple rescaling is generally too restrictive to allow for an accurate description of empirical data. Below we therefore complement the scaling parameters $b_{i}$ by a second parameter affecting also the shape of the survival curve. The resulting CP will be referred to as *generalized scaling CP* or *GS-CP*.

Whereas the implementation of the GS-CP requires one to explicitly estimate the parameters defining the transformations leading from $S_{0}$ to $S_{1}$ and $S_{2}$, the other two CPs are non-parametric. The first is a generalization of the multiplicative null model, which extends the assumption that the relative increases of mean lifespan combine multiplicatively to the entire quantile function Q(s). Here Q(s) denotes the inverse function of the survival curve S(x), that is, Q(s) is the age at which a fraction *s* of the population is still alive. In particular, the median lifespan is given by Q(1/2), and the *generalized multiplicative CP* (*GM-CP*) reads

(2) $$\begin{array}{c} Q_{12}\left(s\right)=\frac{Q_{1}\left(s\right)Q_{2}\left(s\right)}{Q_{0}\left(s\right)}. \end{array}$$

The temporal scaling relation (1) constitutes a special case of (2). We will see below that the transformations underlying the GM-CP can be viewed as inhomogeneous temporal rescalings where the scale factor depends on the fraction of surviving individuals.

In contrast to the GM-CP, which is motivated primarily by formal considerations, the third CP is based on a clear biological picture and can be formally derived within the reliability theory of ageing [29,30]. The key assumption taken from this theory is that the survival of an organism requires the maintenance of several vital functional modules, and the organism dies when one of these modules fails. In the language of failure time analysis the failures of different modules are *competing risks* [31], and independence of longevity interventions implies that they affect disjoint sets of functional modules. A straightforward derivation given below then yields the *competing risks CP* (*CR-CP*)

(3) $$\begin{array}{c} S_{12}\left(x\right)=\frac{S_{1}\left(x\right)S_{2}\left(x\right)}{S_{0}\left(x\right)}. \end{array}$$

Despite the formal similarity between (2) and (3), their implications are markedly different. Firstly, whereas by construction median lifespans combine multiplicatively under the GM-CP (2), and hence standard analysis would detect no interactions, we will show below that the CR-CP (3) contains a generic mechanism for synergistic interaction on the level of median lifespan. Secondly, the requirement that the CR-CP yields a valid combined survival curve $S_{12}\left(x\right)$ poses rather restrictive conditions on the shapes of the survival curves $S_{0}$, $S_{1}$ and $S_{2}$. By contrast, the GM-CP (2) is more easily satisfied.

Below we will explore the mathematical properties of the proposed CPs in more detail and discuss their relation to conventional interaction analysis. We then apply them to a published data set containing measured survival curves for all combinations of four different longevity interventions in *Caenorhabditis elegans*, that is, two genetic mutations, dietary restriction and cold temperature [10]. As each of the six pairs of interventions can occur on four different backgrounds, this data set allows for a total of 24 pairwise analyses. For each pair of interventions, we determine parametrized fits to the four survival curves that are constrained to conform to the CPs and compare them to unconstrained fits. The relative improvement in the accuracy of the fit that is achieved by lifting the constraint can then be interpreted as a measure for the deviation from the specified type of independence. Somewhat surprisingly, we find that most pairs of interventions can be well described by at least one of the CPs. This indicates that the level of interactions, in the general sense defined above, is low. By focusing on cases where one of the possible fits is significantly better than the others, we identify several characteristic patterns that may provide the basis for a classification of the effect of different longevity interventions on the survival curves. Some general conclusions and open problems for future work that follow from our study are outlined in the Discussion.

## 2. Results

### 2.1. Composition Principles

Let $S_{0}$, $S_{1}$, $S_{2}$ and $S_{12}$ be a quadruple of survival curves corresponding to two different interventions, that is, $S_{1}$ and $S_{2}$ result from $S_{0}$ by single interventions and $S_{12}$ results from $S_{0}$ by combining both interventions. We say that this quadruple fulfills a CP if there are mappings $T_{1}$ and $T_{2}$ from the set of survival curves onto itself such that

(4) $$\begin{array}{c} S_{1}=T_{1}\left[S_{0}\right],\;S_{2}=T_{2}\left[S_{0}\right]\;\mathrm{and}\;S_{12}=T_{1}\left[S_{2}\right]=T_{2}\left[S_{1}\right]\;. \end{array}$$

This definition is based on the assumption that longevity interventions can be formally separated from the ageing phenotype on which they act, and that the latter is sufficiently well represented by the survival curve for this approach to be predictive. Although neither of these assumptions is self-evident, the specific examples to be discussed in the following show that the abstract condition (4) unifies several natural conceptualizations of the independence between longevity interventions. It thus provides a useful framework for a generalized, quantitative interaction analysis on the level of survival curves.

The three specific examples of CPs described below are not exhaustive, and indeed it appears to be a major mathematical challenge to classify all possible transformations $T_{i}$ and functions $S_{i}$ for which (4) holds. However, the logic of our approach only requires us to find one CP that is approximately satisfied for a given pair of interventions in order to conclude that interaction, in the broad sense defined here, is absent or at least weak. Finding the specific CP that minimizes the deviation between the non-interacting prediction and the data for a particular case is analogous to (but more complex than) the identification of the proper nonlinear scale on which to measure a phenotype in order to obtain an unbiased estimate of genotypic interactions [11,12,14,32].

### 2.2. Competing Risks CP

The reliability theory of ageing [29,30] uses concepts that were developed in engineering and product design to describe the failure of artificial systems, and applies them to living organisms. The basic idea is that the system can be reduced to a series of blocks, where each block consists of parallel redundant elements, and each element has a certain (constant) failure rate. The blocks in series are interpreted as essential functional modules of an organism, such as organs, which consist of redundant elements, such as cells and pathways. Modules cease to function if all their redundant elements have failed, and the death of the organism is caused by the failure of one of the essential modules.

The key feature of reliability theory that is relevant in the present context is that the probability for the organism to survive up to age *x*, that is, the survival curve S(x), is equal to the product of the probabilities that each of the essential modules is still functional at time *x*. When there are *N* independent modules each characterized by a survival probability $P_{k}\left(x\right)$, the resulting survival curve has the form

(5) $$\begin{array}{c} S\left(x\right)=\prod_{k=1}^{N}P_{k}\left(x\right). \end{array}$$

This mathematical structure is known from failure time analysis as a competing risks model [31]. In this setting the failure of each module *k* is a latent cause of death with its own survivor function $P_{k}$, and the actual time of death or failure is the smallest among the *N* latent failure times. Equation (5) then follows if the risks are independent.

Assuming that a given intervention affects only one of the *N* modules, the corresponding survival probability $P_{k}\left(x\right)$ is replaced by another function $P_{k}^{\prime }\left(x\right)$, which implies the transformation $T_{k}\left[S\right]=S\;\phi_{k}$ with $\phi_{k}\left(x\right)=P_{k}^{\prime }\left(x\right)/P_{k}\left(x\right)$. When two interventions affect different modules, the survival curve corresponding to the combined intervention is then indeed given by $S_{12}=S_{0}\phi_{1}\phi_{2}=S_{1}S_{2}/S_{0}$. If each of the focal interventions affects several of the modules, the CR-CP remains valid provided the two sets of affected modules are disjoint. It is implicit in the product form of (5) that the modules affected by the two interventions are then not only independent of each other, but also independent of all other determinants of lifespan that remain unaffected.

The validity of the CR-CP (3) places rather restrictive conditions on the shapes of the individual survival curves involved. Since both $S_{1}$ and $S_{2}$ are assumed to result from longevity interventions, it is possible that $S_{0}\left(x\right)<S_{1}\left(x\right)S_{2}\left(x\right)$ for large *x*, which would lead to a violation of the condition that $S_{12}\left(x\right)\leq 1$. For a quantitative analysis of the conditions of validity of (3) we consider Weibull survival curves of the form

(6) $$\begin{array}{c} S_{i}\left(x\right)=\exp\left(-a_{i}x^{n_{i}}\right) \end{array}$$

with positive parameters $a_{i}$ and $n_{i}$ for i=0,1,2. Constructing the double-intervention survival curve yields

(7) $$\begin{array}{c} S_{12}\left(x\right)=\exp\left[-a_{1}x^{n_{1}}-a_{2}x^{n_{2}}+a_{0}x^{n_{0}}\right]\;. \end{array}$$

It is easy to see that a necessary condition for the combined curve to be monotonically decreasing is that $\min\left[n_{1},n_{2}\right]\leq n_{0}\leq \max\left[n_{1},n_{2}\right]$. Setting $n_{0}=n_{1}=n_{2}=n$ the combined survival curve is again of Weibull form, but it is valid only if $a_{0}<a_{1}+a_{2}.$ In terms of the median lifespans $m_{i}={\left(\ln2/a_{i}\right)}^{\frac{1}{n}}$, this condition reads

(8) $$\begin{array}{c} {\left(\frac{m_{0}}{m_{1}}\right)}^{n}+{\left(\frac{m_{0}}{m_{2}}\right)}^{n}>1. \end{array}$$

If both interventions are of equal effect, $m_{1}/m_{0}\approx m_{2}/m_{0}$, this condition can be satisfied only if this effect is rather weak, $m_{1}/m_{0}<2^{1/n}$ where often n≫1 [22]. On the other hand, the condition (8) can also be satisfied by interventions of widely different effects, for example, $m_{1}/m_{0}\approx 1$ and $m_{2}/m_{0}\gg 1$. When the condition (8) is satisfied, the median lifespan of the combined intervention is given by

(9) $$\begin{array}{c} m_{12}={\left(m_{1}^{-n}+m_{2}^{-n}-m_{0}^{-n}\right)}^{-1/n}, \end{array}$$

which can be shown to always exceed the multiplicative expectation (see Appendix A.1 for the derivation). Thus, at least for the simple case of Weibull survival curves with equal index *n*, the CR-CP predicts positive (synergistic) interaction for median life spans, and it is expected to hold preferentially for interventions of strongly unequal effect. We believe that this conclusion holds also beyond the particular class of Weibull curves, and we will see below that the pattern described is indeed found in empirical data.

Finally, we note that the CR-CP takes a simple form when written in terms of the age-dependent mortalities or hazard rates defined by $h_{i}=-\left(1/S_{i}\right)\;dS_{i}/dx$. Indeed, using (3), it follows that $h_{12}=h_{1}+h_{2}-h_{0}$ or

(10) $$\begin{array}{c} h_{0}-h_{12}=h_{0}-h_{1}+h_{0}-h_{2}, \end{array}$$

which implies that the reductions of mortality afforded by the two focal interventions add up in the combination.

### 2.3. Generalized Multiplicative CP

The basic assumption underlying this principle is that the age x=Q(s) at which a certain fraction *s* of individuals is still alive is multiplied by an *s*-dependent factor $f_{i}\left(s\right)$ in the presence of an intervention *i*. In particular, the median lifespan $m_{0}=Q_{0}\left(1/2\right)$ of the baseline population would be multiplied by $f_{i}\left(1/2\right)$. In terms of survival curves, the intervention results in the multiplication of the inverse survival curve with the function $f_{i}$, that is, $T_{i}\left[S\right]={\left(S^{-1}\;f_{i}\right)}^{-1}\;,$ where $F^{-1}$ denotes the inverse of a function *F*. The survival curve corresponding to the double-intervention can then be written as

(11) $$\begin{array}{c} S_{12}={\left(S_{0}^{-1}f_{1}f_{2}\right)}^{-1}={\left(\frac{S_{1}^{-1}\;S_{2}^{-1}}{S_{0}^{-1}}\right)}^{-1}\;, \end{array}$$

which is equivalent to (2). By construction, validity of the GM-CP ensures that median lifespans combine multiplicatively,

(12) $$\begin{array}{c} m_{12}=\frac{m_{1}m_{2}}{m_{0}}, \end{array}$$

because $m_{i}=Q_{i}\left(1/2\right)$.

Equation (2) implies that the GM-CP is fulfilled if $S_{0}$, $S_{1}$ and $S_{2}$ are chosen arbitrarily and $S_{12}$ is constructed according to the right-hand side of (11). However, similar to the situation described above for the CR-CP, the resulting curve $S_{12}$ may not be a valid survival curve. To see this, we consider again the example of the Weibull survival curve (6). The inverse function reads $Q_{i}\left(s\right)={\left(-\log\left(s\right)/a_{i}\right)}^{1/n_{i}}$, and after some algebra one finds that the combined survival curve is again of Weibull form, $S_{12}=\exp\left(-a_{12}\;x^{n_{12}}\right)$, with $a_{12}={\left({a_{1}}^{1/n_{1}}{a_{2}}^{1/n_{2}}{a_{0}}^{-1/n_{0}}\right)}^{n_{12}}$ and $n_{12}={\left({n_{1}}^{-1}+{n_{2}}^{-1}-{n_{0}}^{-1}\right)}^{-1}$. Since $S_{12}$ is a valid survival curve only if $n_{12}>0$, the condition on the parameters is $n_{1}^{-1}+n_{2}^{-1}>n_{0}^{-1}$.

### 2.4. Generalized Scaling CP

Rather than multiplying a survival curve or its inverse with a function, one can also think of applying a function to a survival curve S(x) (outer scaling) or to its argument *x* (temporal scaling). This yields a transformation of the general form $T_{i}\left[S\right]\left(x\right)=g_{i}\left(S\left(t_{i}\left(x\right)\right)\right)$. In order to ensure the validity of the general CP (4), the functions $g_{i}$ and $t_{i}$ have to fulfill the conditions $g_{1}\left(g_{2}\left(x\right)\right)=g_{2}\left(g_{1}\left(x\right)\right)$ and $t_{1}\left(t_{2}\left(x\right)\right)=t_{2}\left(t_{1}\left(x\right)\right)$, respectively, for all *x*. Furthermore, the functions have to preserve the survival curve properties and hence $g_{i}\left(0\right)=t_{i}\left(0\right)=0$, $g_{i}\left(1\right)=1$ and $t_{i}\left(x\to \infty \right)=\infty$.

A simple choice that satisfies all these conditions is a linear scaling of time [28], $t_{i}\left(x\right)=b_{i}x$, and a power function applied to the survival curve, $g_{i}\left(s\right)=s^{q_{i}}$, with positive constants $b_{i}$ and $q_{i}$. Starting from a baseline survival curve $S_{0}$, the single-intervention curves are then of the form

(13) $$\begin{array}{c} S_{i}\left(x\right)=S_{0}{\left(b_{i}x\right)}^{q_{i}},\;\;i=1,2. \end{array}$$

In terms of the hazard rates, Equation (13) takes the form $h_{i}\left(x\right)=q_{i}b_{i}h_{0}\left(b_{i}x\right)$. This shows that the transformation combines an accelerated failure rate model (parametrized by $b_{i}$) with a proportionate risk model (parametrized by $q_{i}$) [28,31]. The *generalized scaling CP* (*GS-CP*) is satisfied if constants $b_{1},b_{2},q_{1},q_{2}$ can be found such that the survival curve of the combined intervention is given by

(14) $$\begin{array}{c} S_{12}\left(x\right)=S_{0}{\left(b_{1}b_{2}\;x\right)}^{q_{1}q_{2}}. \end{array}$$

As was mentioned above, in the case of purely temporal scaling ($q_{1}=q_{2}=1$), the transformed curves satisfy the GM-CP (2), but in general the GS-CP does not reduce to any of the other two CPs. For the special case when $S_{0}$ is of Weibull form (6), the transformation (13) amounts to a pure temporal rescaling with scale factor $q_{i}^{1/n_{0}}b_{i}$. Correspondingly, the median lifespans combine multiplicatively, as in (12), under the GS-CP. However, for survival curves of Gompertz form, the GS-CP is consistent with both antagonistic and synergistic interaction on the level of the most likely lifespan (see Appendix A.2 for details).

### 2.5. Data Set

As an illustration of our approach, we analyzed a published data set for *C. elegans* exposed to four different longevity interventions [10]. These included two genetic mutations (*clk-1* and *daf-2*), cold temperature (16 °C vs. 25 °C at control conditions) and dietary restriction (axenic medium). Survival curves were obtained in triplicate for each of the $2^{4}=16$ possible combinations of interventions. In order to achieve the large cohort sizes required for a meaningful fit of survivorship data to survival functions [21,22], we pooled the replicates for each set of conditions, which yields cohorts of more than 300 individuals. Since each of the six pairs of interventions can be applied to four different baseline conditions including zero, one or two other interventions, the data allow for 24 different pairwise comparisons. Each comparison makes use of a quadruple of survival curves comprising the baseline condition, each of the focal interventions applied individually, and the combination of the two focal interventions.

For a better overview of the relation between survival curves, we assign a binary string to each of them. A position of the string corresponds to a certain intervention, with a 0/1 at this position determining whether the corresponding intervention takes place. The assignment of interventions is as follows: The first position indicates reduced temperature, the second the *daf-2* mutation, the third the *clk-1* mutation and the fourth position corresponds to dietary restriction. For example, the string 1001 labels the survival curve at 16 °C with dietary restriction but in the absence of genetic mutations. In this notation, a quadruple of survival curves is represented by two strings that differ at two positions and the two intermediate strings that differ in one position from either of the two aforementioned strings. A valid quadruple would be, for instance, 1001 (baseline), 1101, 1011 and 1111. For the sake of brevity we will write 1001–1111 for this quadruple of survival curves. The full list of combinations of interventions is given in Table 1.

### 2.6. Test of Composition Principles

To quantify the consistency of the empirical data with the proposed CPs, we compare the quality of a fit constrained to satisfy a given CP with that of an unconstrained fit. All fits are based on 3-parameter survival functions of the form

(15) $$\begin{array}{c} S_{i}\left(x\right)={\left[1-{\left\{1-\exp\left(-\mu_{i}x\right)\right\}}^{M_{i}}\right]}^{N_{i}}. \end{array}$$

Within reliability theory, the parameters of (15) are interpreted as the failure rate of redundant elements $\mu_{i}$, the number of redundant elements $M_{i}$ and the number of essential functional modules $N_{i}$ [30]. We should like to emphasize, however, that our use of this particular functional form in the present context is motivated solely by the observation that it is sufficiently versatile to provide satisfactory fits to a wide range of empirical survival curves using a moderate number of parameters. The parameters $M_{i}$ and $N_{i}$ will therefore not be constrained to take on integer values. To verify that our conclusions do not depend on the particular family of survival functions that is used to implement the analysis, we have carried out a second set of fits using a three-parameter logistic mortality model [24]. The exemplary results shown in Appendix B are indistinguishable from those based on (15).

The fit algorithm described in the Materials and Methods section minimizes the sum of squares of the mean square deviations (SSD) corresponding to the four curves in the quadruple

(16) $$\begin{array}{c} D=D_{0}^{2}+D_{1}^{2}+D_{2}^{2}+D_{12}^{2}\;, \end{array}$$

where $D_{i}=\frac{1}{k_{i}}\sum_{x=0}^{k_{i}}{\left(S_{i}\left(x\right)-{\tilde{S}}_{i}\left(x\right)\right)}^{2}$ with ${\tilde{S}}_{i}\left(x\right)$ denoting the empirical surviving fraction and $k_{i}$ the number of data points. In the first step of the analysis, the survival curves are fitted individually, which implies that the terms in (16) are independent. The resulting optimal SSD is denoted by $D_{\mathrm{ind}}$. In the next step, a second fit is carried out under the constraint imposed by the CP of interest. The implementation of this step differs between the different CPs introduced above.

- A direct fitting algorithm constrained to satisfy the CR-CP (3) will in most cases fail to converge to a valid survival curve. This reflects the restrictive conditions on the individual curves imposed by this CP. To overcome this difficulty, we further constrained the fitting procedure by demanding that the four survival curves in the quadruple take the specific form
  (17) $$\begin{array}{c} S_{0}=F_{1}F_{2}F_{3},\;S_{1}=F_{1}F_{3},\;S_{2}=F_{2}F_{3},\;S_{12}=F_{3}, \end{array}$$
  where the $F_{i}\left(x\right)$ are again represented by three-parameter functions (15). This enforces the validity of the CR-CP (3) but also implies that the curves have to be ordered according to $S_{12}\left(x\right)\geq S_{1}\left(x\right),S_{2}\left(x\right)\geq S_{0}\left(x\right)$ for all *x*.
- For the GM-CP, the survival curves $S_{0}$, $S_{1}$ and $S_{2}$ are represented by three survival functions of the form (15), and the fourth curve $S_{12}$ is constructed according to (11) using the numerical computation of inverse functions. The nine parameters entering the three functions are then adjusted to optimize the fit to the data quadruple.
- Finally, for the implementation of the GS-CP, the fit determines a single three-parameter survival function $S_{0}\left(x\right)$ along with the four parameters $b_{1},b_{2},q_{1},q_{2}$ entering the scaling transformations (13) and (14).

Note that different quadruples have in general a different inherent difficulty to be fitted. As we are interested primarily in the relative quality of the constrained fits associated with different CPs, we normalize the SSD *D* for fits that fulfil a CP by the SSD $D_{\mathrm{ind}}$ obtained when the four curves are fitted independently. Doing this enables us to assess how well the different CPs are satisfied for different quadruples of data. It turns out that the independent fits to the three-parameter survival function (15) yield accurate approximations to the measured survival curves in all cases. Moreover, all 24 quadruples of survival curves can be fitted reasonably well by at least one of the three CPs.

Examples of three experimental quadruples and the corresponding fits are shown in Figure 1. For each column, a different type of CP yields the lowest relative SSD. In column (a), the CR-CP provides the best quality of the fit, in column (b) it is the GM-CP, and the GS-CP in column (c). In all three cases the relative SSD $D/D_{\mathrm{ind}}$ of the best fit is very close to unity, showing that the corresponding CP is satisfied with high accuracy. A full set of figures showing all pairs of empirical survival curves with their respective optimal fits can be found in the Supplementary Material (Figures S1–S24).

It is evident that the examples shown in the three columns represent different patterns. Column (a) depicts the interaction of the *clk-1* mutation with DR at low temperature. The effect of *clk-1* on lifespan is hardly detectable in the absence of DR but becomes significant when DR is applied as well. This provides an example of synergistic interaction for mean lifespan between two interventions of widely different individual effects. As we have seen that apparent synergistic interaction between interventions of strongly unequal effects is a generic feature of the CR-CP, it is not surprising that this CP is able to describe these data very well. By contrast, the survival curves in column (b) show two interventions of similar effect (low temperature and DR applied to the *daf-2* mutant) which combine essentially multiplicatively in terms of mean lifespan. Since the GM-CP satisfies multiplicativity of the median lifespan by construction, it yields the best fit to the data in this case. Finally, column (c) displays a case of apparent antagonistic interaction, where the combined interventions of the *clk-1* mutation and DR on the background of low temperature and *daf-2* are essentially indistinguishable from the effects of the individual interventions. The GS-CP is the only one of the three CPs that is principally able to account for antagonistic interaction for lifespan, and therefore it provides the best description of these data.

**Figure 1:**
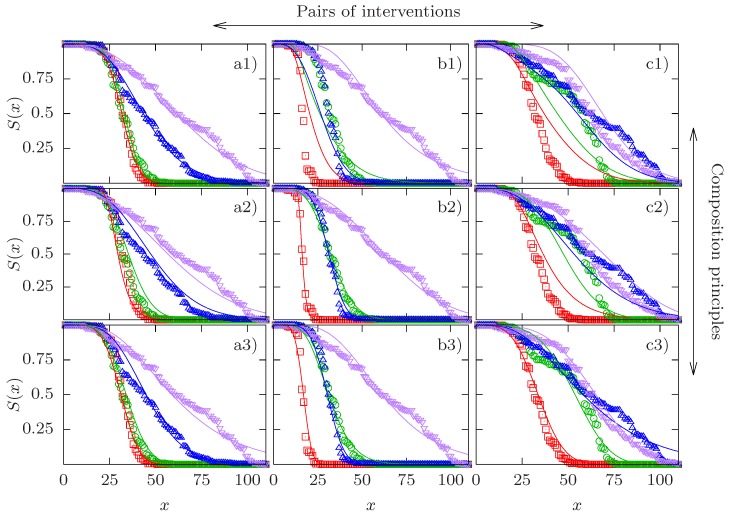
Comparison of experimental survival curves and model fits for three cases. Experimental survival curves are depicted by symbols and their respective fits by lines. Columns correspond to different pairs of interventions and rows correspond to different composition principles (CPs). Column a) shows the quadruple 1000–1011, column b) the quadruple 0010–1011, and column c) the quadruple 1010–1111. Row 1) shows the competing risks CP, row 2) shows the generalized multiplicative CP, and row 3) shows the generalized scaling CP. Red squares represent the baseline curve, green circles and blue upward triangles display the two single interventions in the order of their position in the binary string (green circles first, blue upward triangles second), and purple downward triangles correspond to the combined interventions. The fits in panels a1), b2) and c3) have the best quality in their respective column in terms of their sum of squared deviations *D* defined in (16). The relative SSDs of the three best fits are $D/D_{\mathrm{ind}}=0.834$ (a1), 1.06 (b2) and 1.06 (c3).

**Figure 2:**
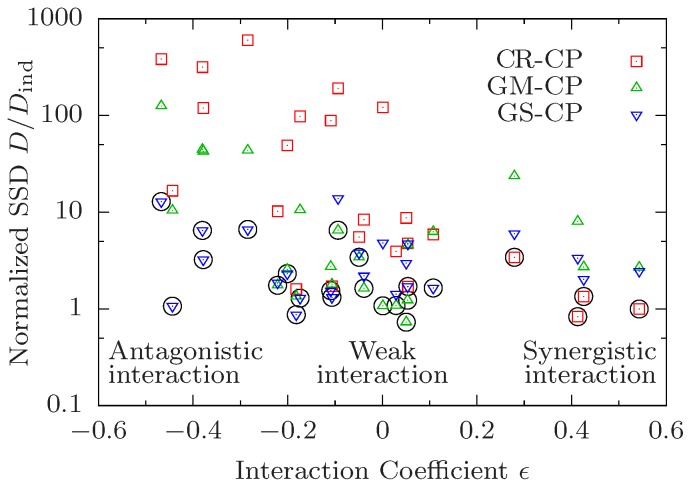
Preference for different composition principles correlates with interaction in median lifespan. The sum of squared deviations *D* of survival curves satisfying a composition principle (CP) is divided by the SSD $D_{\mathrm{ind}}$ of independently fitted curves and shown in dependence on the median interaction coefficient ϵ defined in (18). Each symbol corresponds to a combination of a data quadruple and a CP. The CP yielding the best result for a given quadruple is marked by a black circle.

The correlation between the preferred CP and the type of interaction on the level of median lifespan that is observed in the examples shown in Figure 1 holds quite generally across all 24 pairwise comparisons. In Figure 2 we plot the ratio $D/D_{\mathrm{ind}}$ vs. the interaction coefficient of median lifespans defined as

(18) $$\begin{array}{c} \epsilon =\frac{m_{0}\;m_{12}}{m_{1}\;m_{2}}-1\;. \end{array}$$

The interaction coefficient vanishes under the multiplicative condition (12), and is positive (negative) in the presence of synergistic (antagonistic) interaction. Figure 2 thus illustrates the relationship between interaction for median lifespan quantified by ϵ, and interaction on the level of survival curves quantified by the minimal value of $D/D_{\mathrm{ind}}$. As discussed previously, survival curves obeying the CR-CP tend to favour synergistic interaction for median lifespan and hence there is a negative correlation between the median interaction coefficient ϵ and the normalized SSD $D/D_{\mathrm{ind}}$ for this CP (red squares in Figure 2). In the same manner, curves obeying the GM-CP display a lower goodness of fit (larger relative SSD) the larger the absolute value |ϵ|. As there is no a-priori preference of the GS-CP for a particular type of interaction, fits performed under this principle yield decent results for all values of ϵ. Accordingly, looking only at the CP that yields the best result for a given data quadruple, one observes that the CR-CP works best for data with strong synergistic interaction while GM-CP works best when interaction is weak. Because both principles perform poorly with strong antagonistic interaction, the GS-CP yields the lowest SSD in this regime.

Apart from this conspicuous pattern, however, the most striking feature of Figure 2 is that interaction on the level of survival curves is remarkably weak, in the sense that the minimal value $\delta \equiv \min_{\mathrm{CP}}\left\{D/D_{\mathrm{ind}}\right\}$ is often close to unity. Specifically, δ<2 in 16 out of 24 cases, and there is only one quadruple (0000–1100, see Figure S1) for which δ>10. The latter corresponds to the combination of *daf-2* and low temperature, which was found to display significant antagonistic interactions for mean lifespan in the original work of Yen and Mobbs [10]. These authors also observed negative interactions between *daf-2* and dietary restriction. In our analysis we find that these interventions interact strongly in the presence of *clk-1* (quadruple 0010–0111, Figure S18, has δ=6.54) but not on the control background (quadruple 0000–0101, Figure S17, has δ=1.28). The interaction for median life span is significant and negative in both cases.

Altogether, three out of the four quadruples with δ>5 comprise one of the two pairs of interacting interventions identified in [10] on different backgrounds. The fourth corresponds to the combination of dietary restriction and cold temperature (0000–1001, Figure S13) for which the interaction for median lifespan is weak (ϵ=−0.09). On the other hand, the quadruples 1000–1011 [Figure 1a) and Figure S23] and 1010–1111 [Figure 1c) and Figure S20] display significant positive (ϵ>0.4) and negative (ϵ<−0.4) interaction for median lifespan, respectively, but both have δ≈1. Overall, Figure 2 makes it evident that interaction for median lifespan is a poor predictor for the existence of interactions on the level of the survival curves.

## 3. Discussion

The composition principles introduced above quantify different natural notions of independence between longevity interventions. The GM-CP generalizes the commonly used multiplicative model for relative life span increases to the quantile function Q(s), which is sufficient to predict the survival curve of the combined intervention from the survival curves representing the individual interventions. The CR-CP follows under rather general conditions from a modular structure of the functions on which the survival of the organism depends, as exemplified by (but not restricted to) the reliability theory of ageing. Finally, the GS-CP is based on the assumption that longevity interventions can be viewed as generalized scaling transformations applied to the survival curve, which are commutative and therefore yield a unique prediction for the combined survival curve.

Two of the three CPs (GM and CR) are non-parametric, in the sense that they can be formulated without reference to a particular parametrization of the survival curves $S_{i}$ or the longevity transformations $T_{i}$. One might have expected that this property would facilitate the application of these CPs to data, but this is in fact not the case. The direct test of the CR-CP is considerably exacerbated by the fact that the insertion of an arbitrary set of survival functions $S_{0},S_{1}$ and $S_{2}$ on the right-hand side of (3) does not generally produce a valid survival curve. Similar problems may arise for the GM-CP (2). In comparison, the application of the parametric GS-CP is more straightforward. In addition, it has the benefit of yielding some insight into the nature of the longevity transformations involved through the estimates of the parameters $b_{i}$ and $q_{i}$ in (13). As we have outlined above, the CR-CP and the GS-CP have natural interpretations in terms of the competing risks, proportionate hazard and accelerated failing rate models of survival analysis [31].

An important conclusion from our approach is that independence of longevity interventions on the level of survival curves does not generally imply the absence of interaction for median lifespan. This point is most clearly illustrated by the CR-CP, which is based on a biologically plausible concept of independence in terms of modularity of vital functions, and implies additivity of age-dependent mortality in the sense of (10). Nevertheless, as we have demonstrated for a class of survival functions, interventions combined according to the CR-CP can display substantial synergistic interaction in their effect on lifespan. We believe that this is true irrespective of the specific form of the survival curve, and a proof of this conjecture would be of considerable interest. For the GS-CP we have shown that the apparent interaction for the most likely lifespan can be positive or negative depending on the parameters entering the longevity transformation.

Our explorative investigation of the empirical data set of [10] shows that all quadruples of survival curves can be fitted rather well by at least one of the CPs. This indicates that "true" interactions that would become manifest in a violation of the general composition principle (4) are rare, even though interaction for median lifespan can be quite significant (see Figure 2). It remains to be seen if this outcome is specific to the data set under investigation. None of the three suggested types of CPs were found to be universally preferred. Instead, the preference for a given CP is correlated with the amount and sign of interaction on the level of median lifespan. In this way, our analysis decomposes the 24 pairs of survival curves into three classes with qualitatively different patterns of interactions. So far we have not been able to clearly attribute individual pairs of interventions to specific classes. With one exception (the combination of low temperature and *daf-2*, which always falls into the GS class), the attribution generally varies according to the identity of the two background interventions.

Moreover, despite our pooling of data obtained from different experiments, the attribution appears to be significantly affected by measurement error. This is illustrated in Appendix C, where we show the results of an analysis using single-set survival experiments corresponding to the largest cohort size. Although the overall pattern is similar to Figure 2, the attribution of specific pairs of interventions to their preferred CPs differs considerably and the correlation with the interaction parameter ϵ is weakened. We expect that the recently developed methods for the generation of high-resolution survival curves [33,34] will help to alleviate this problem and allow one to extract specific functional information from the kind of analyses proposed here.

## 4. Materials and Methods

The fitting algorithm aims to minimize the sum of squared deviations *D* defined in (16). Even though the survival curves occurring in this paper have a relatively simple shape, it is still rather difficult to fit several interdependent curves at once. In particular, standard hill-climbing algorithms tend to converge to suboptimal minima of *D*. We therefore use an evolutionary algorithm that consists of the following steps:

1. The algorithm is initialized with a population of *n* quadruples of survival functions. Initial parameter values are $\mu_{i}=M_{i}=N_{i}=b_{i}=q_{i}=1.0$.
2. Next, *m* offspring are created that descend from randomly chosen parents. The parameters of the children are equal to the parents’ parameters multiplied with a factor $e^{uX}$, where *X* is uniform random variable on [−1,1] and u>0 is the mutation strength.
3. Out of the total population of the n+m individuals, the *n* with lowest SSD survive. These individuals make up the next generation.
4. Mutation strength *u* is decreased by a constant factor, and the algorithm continues with the second step.

For the fits in this paper we chose n=m=180 and ran the algorithm for 2500 generations. We chose u=1 for the initial generation and decreased it in every generation by a constant factor such that u=0.01 in the final generation. The solutions obtained in this way generally provided good approximations of the empirical data. Because of the high dimensionality of the parameter space, however, there is no guarantee that the algorithm converges to the true optimum of the cost function (16). Since the constraints due to the CPs reduce the dimension of the parameter space, this can occasionally lead to situations where the constrained fit is somewhat better than the unconstrained one, $D/D_{\mathrm{ind}}<1$.

## Figures and Tables

**Table 1:** Binary representation used to label combinations of longevity interventions in the data set of Yen and Mobbs [10].

Intervention	Binary	Intervention	Binary
None/control	0000	Dietary Restriction (DR)	0001
16 °C	1000	DR at 16 °C	1001
*daf-2*	0100	*daf-2* & DR	0101
*daf-2* at 16 °C	1100	*daf-2* & DR at 16 °C	1101
*clk-1*	0010	*clk-1* & DR	0011
*clk-1* at 16 °C	1010	*clk-1* & DR at 16 °C	1011
*clk-1* & *daf-2*	0110	*clk-1*& *daf-2* & DR	0111
*clk-1* & *daf-2* at 16 °C	1110	*clk-1* & *daf-2* & DR at 16 °C	1111

**Figure A1:**
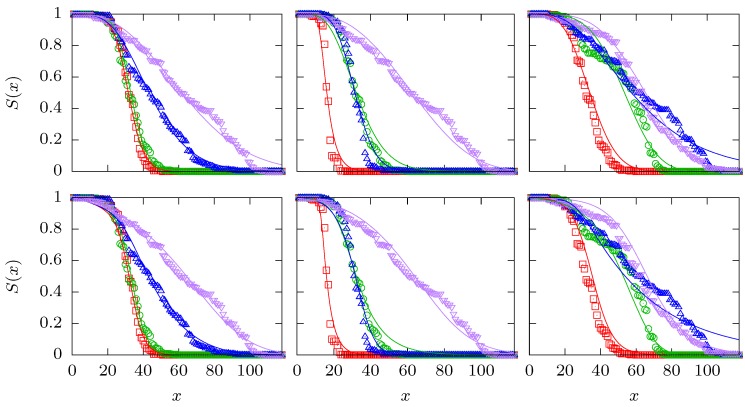
The upper row of panels shows the optimal fits for the quadruples 1000–1011, 0010–1011 and 1010–1111 that are also displayed in the panels (a1), (b2) and (c3) of Figure 1. The lower row shows the corresponding optimal fits using the logistic function (A9) in conjuction with the same three CPs (CR, GM and GS).

**Figure A2:**
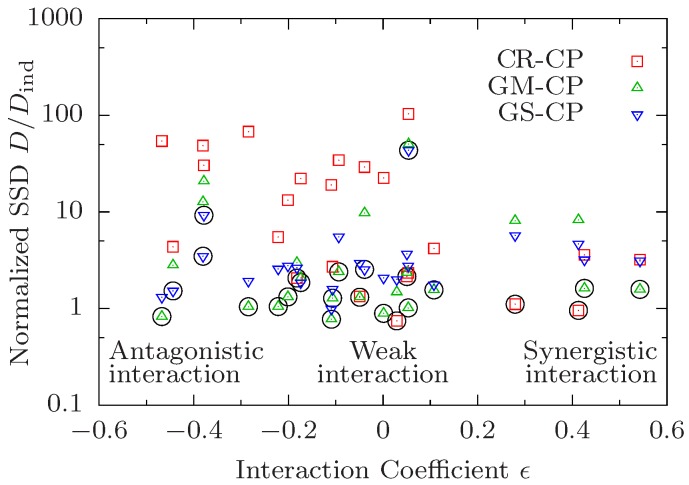
The sum of squared deviations *D* of survival curves satisfying a composition principle (CP) is divided by the SSD $D_{\mathrm{ind}}$ of independently fitted curves and shown in dependence on the median interaction coefficient ϵ defined in Equation (18). Each symbol corresponds to a combination of a data quadruple and a CP. The CP yielding the best result for a given quadruple is marked by a black circle.

## Supplementary Materials

The following are available online at www.mdpi.com/2079-7737/7/1/6/s1, Figures S1–S24: Optimal fits obtained under the three composition principles for each of the 24 quadruples of survival curves.

## Abbreviations

The following abbreviations are used in this manuscript:

CP	Composition principle
CR-CP	Competing risks CP
GM-CP	Generalized multiplicative CP
GS-CP	Generalized scaling CP
SSD	Sum of squares of mean square deviations

## Appendix A. Interaction for Median and Most Likely LifeSpan

Here we demonstrate in two simple cases how apparent interaction for mean or median lifespan, defined as a deviation from the multiplicative expectation, can arise from the validity of a CP.

### Appendix A.1. CR-CP Applied to Weibull Survival Curves

We assume that the baseline survival curve $S_{0}$ and the two single intervention survival curves $S_{1,2}\left(x\right)$ are all of Weibull form with the same index *n*. Then the combined survival curve is of the same form and the median lifespan of the combined intervention is predicted by the CR-CP to be given by Equation (11) of the main text, which reads

(A1) $$m_{12}={\left(m_{1}^{-n}+m_{2}^{-n}-m_{0}^{-n}\right)}^{-1/n}.$$

To prove that this implies ϵ>0, we multiply both sides by $m_{0}/\left(m_{1}m_{2}\right)$ yielding

(A2) $$\epsilon +1=\frac{m_{12}m_{0}}{m_{1}m_{2}}={\left[{\left(\frac{m_{1}}{m_{0}}\right)}^{n}+{\left(\frac{m_{2}}{m_{0}}\right)}^{n}-{\left(\frac{m_{1}m_{2}}{m_{0}^{2}}\right)}^{n}\right]}^{-1/n}.$$

Setting ${\left(m_{1}/m_{0}\right)}^{n}=1+a$ and ${\left(m_{2}/m_{0}\right)}^{n}=1+b$ with a,b>0, the expression inside the square brackets becomes

(A3) 1+a+1+b−(1+a)(1+b)=1−ab<1.

Thus the right hand side of (A2) is greater than unity and ϵ>0. Since mean and median lifespan for the Weibull function (6) only differ by an *n*-dependent factor, these results hold for mean lifespan as well.

### Appendix A.2. GS-CP Applied to Gompertz Survival Curves

Here we assume that all survival curves are of Gompertz form [24]

(A4) $$S\left(x\right)=\exp\left[-\frac{A}{G}\left(e^{Gx}-1\right)\right]$$

with parameters A,G>0. The corresponding hazard rate is $h\left(x\right)=Ae^{Gx}$. The rescaling transformation defined in Equation (13) of the main text changes the parameters $A_{0}$, $G_{0}$ of the baseline survival curve into

(A5) $$A_{i}=q_{i}b_{i}A_{0},\;\;G_{i}=b_{i}G_{0},\;\;\;i=1,2.$$

For calculational convenience, we use the most likely life span $\tilde{m}$ rather than the median life span to characterize the effect of this transformation on longevity. Since −dS/dx is the probability density of lifespan, the most likely lifespan satisfies the equation $d^{2}S/dx^{2}=0$, which in the case of Gompertz survival curves implies

(A6) $$\tilde{m}=G^{-1}\ln\left(G/A\right).$$

Under the transformation (A5) the most likely lifespan changes from ${\tilde{m}}_{0}$ into

(A7) $${\tilde{m}}_{i}=\frac{1}{b_{i}}{\tilde{m}}_{0}+\frac{1}{b_{i}G_{0}}\ln\left(1/q_{i}\right).$$

Thus the temporal rescaling by a factor of $1/b_{i}$ is complemented by an additive correction, the sign of which depends on whether $q_{i}>1$ or $q_{i}<1$. Under the GS-CP, the parameters of the combined longevity transformation are $b_{12}=b_{1}b_{2}$ and $q_{12}=q_{1}q_{2}$. It follows that the deviation of the combined most likely lifespan from the multiplicative expectation is given by

(A8) $$\tilde{\epsilon }\equiv {\tilde{m}}_{12}{\tilde{m}}_{0}-{\tilde{m}}_{1}{\tilde{m}}_{2}=-\frac{1}{b_{1}b_{2}G_{0}^{2}}\ln\left(1/q_{1}\right)\ln\left(1/q_{2}\right),$$

which can be positive or negative depending on the relative signs of $\ln(1/q_{1})$ and $\ln(1/q_{2})$.

## Appendix B. Alternative Fits Using a Logistic Model

To show that the quality of the fits is independent of the model used to fit the data, in Figure A1 we compare the best combined fits obtained using the three-parameter survival function of Equation (15) of the main text to the best combined fits using a three-parameter logistic model [24]. The logistic model is defined by the survival function

(A9) $$S\left(x\right)={\left[1+s\frac{A}{G}\left(e^{Gx}-1\right)\right]}^{-1/s}.$$

For s→0 this reduces to the standard Gompertz law with exponentially growing mortality $h\left(x\right)=Ae^{Gx}$, and the parameter s>0 induces a saturation of mortality at a limiting value of G/s.

## Appendix C. Interaction Analysis for a Single Set of Survival Curves

The survival curves used for the analysis in the main text were obtained by pooling the survivorship data from three independent replicates of the experiment carried out by Yen and Mobbs [10]. The following Figure A2 shows the corresponding result (analogous to Figure 2) obtained by using a single replicate with the largest cohort size of 150 individuals.
