# Supplementary material for: Interaction Analysis of Longevity Interventions Using Survival Curves
Source: Biology (Basel). 2018 Jan 6;7(1):6. doi: 10.3390/biology7010006 (PMC5872032; doi:10.3390/biology7010006)
Supplement: Supplementary file 1 [file biology-07-00006-s001.pdf]

Supplementary material

S. Nowak, J. Neidhart, J. Rzezonka, I.G. Szendro, R. Marathe, J. Krug

December 20, 2017

The following figures S1-S24 display the optimal fits obtained under the three composition principles for each of the 24 quadruples of survival curves. Red squares represent the baseline curve, green circles and blue upward triangles display the two single interventions in the order of their position in the binary string (green circles first, blue upward triangles second), and purple downward triangles correspond to the combined interventions. The resulting fit parameters and SSD's are given in the captions. Fits with lowest SSD are marked with a circled asterisk in the corresponding figure panel. The ordering of the fit parameters corresponds to that of the survival curves. That is, μ_0, M_0, N_0 are the parameters of the baseline survival curve S_0, μ_1, M_1, N_1 and b_1, q_1 belong to the first single intervention and μ_2, M_2, N_2 and b_2, q_2 belong to the second single intervention.

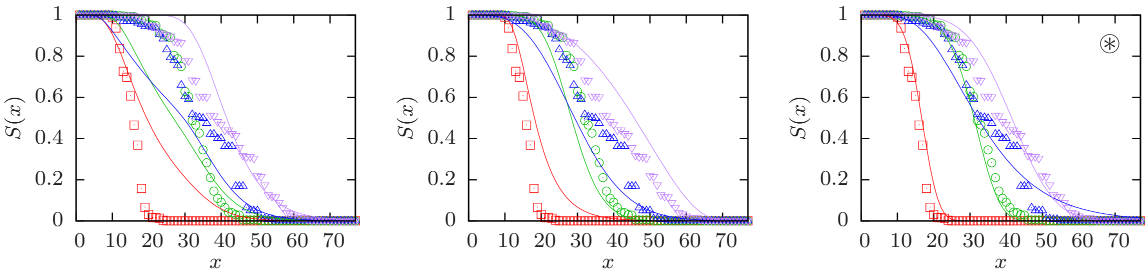

(a) Fit of $S(x)$ obeying CR-CP. SSD $= 1794 \cdot 10^{-7}$. Parameters: $\mu_1 = 0.115$; $M_1 = 104.703$; $N_1 = 1.345$; $\mu_2 = 0.466$; $M_2 = 34.758$; $N_2 = 0.064$; $\mu_3 = 0.306$; $M_3 = 47.294$; $N_3 = 0.158$.

(b) Fit of $S(x)$ obeying GM-CP. SSD $= 588 \cdot 10^{-7}$. Parameters: $\mu_1 = 0.287$; $M_1 = 73.246$; $N_1 = 0.625$; $\mu_2 = 0.072$; $M_2 = 15.565$; $N_2 = 5.548$; $\mu_3 = 0.026$; $M_3 = 4.377$; $N_3 = 10.272$.

(c) Fit of $S(x)$ obeying GS-CP. SSD $= 60 \cdot 10^{-7}$. Parameters: $\mu_1 = 0.039$; $M_1 = 7.333$; $N_1 = 157.411$; $b_1 = 0.258$; $q_1 = 69.069$; $b_2 = 1.736$; $q_2 = 0.008$.

Figure S1. Quadruple 0000-1100. $\epsilon = -0.4670$, $D_{\text{ind}} = 4.685 \cdot 10^{-7}$.

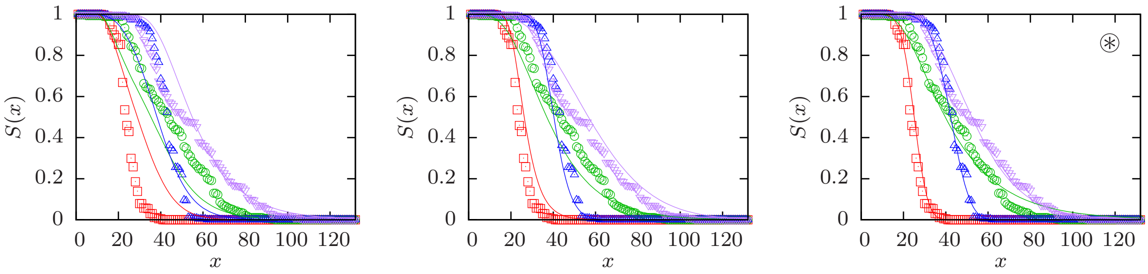

(a) Fit of $S(x)$ obeying CR-CP. SSD $= 1471 \cdot 10^{-7}$. Parameters: $\mu_1 = 0.095$; $M_1 = 74.923$; $N_1 = 0.628$; $\mu_2 = 0.055$; $M_2 = 8.276$; $N_2 = 1.434$; $\mu_3 = 0.431$; $M_3 = 423.478$; $N_3 = 0.07$.

(b) Fit of $S(x)$ obeying GM-CP. SSD $= 207 \cdot 10^{-7}$. Parameters: $\mu_1 = 0.186$; $M_1 = 94.905$; $N_1 = 1.024$; $\mu_2 = 0.056$; $M_2 = 5.862$; $N_2 = 1.168$; $\mu_3 = 0.095$; $M_3 = 80.124$; $N_3 = 3.548$.

(c) Fit of $S(x)$ obeying GS-CP. SSD $= 30 \cdot 10^{-7}$. Parameters: $\mu_1 = 0.053$; $M_1 = 12.033$; $N_1 = 27.461$; $b_1 = 1.858$; $q_1 = 0.016$; $b_2 = 0.487$; $q_2 = 3.43$.

Figure S2. Quadruple 0001-1101. $\epsilon = -0.3802$, $D_{\text{ind}} = 4.650 \cdot 10^{-7}$.

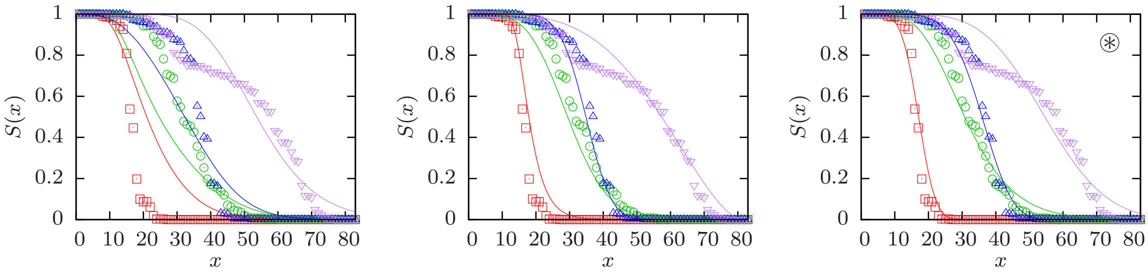

(a) Fit of $S(x)$ obeying CR-CP. SSD $= 2218 \cdot 10^{-7}$. Parameters: $\mu_1 = 0.046$; $M_1 = 17.589$; $N_1 = 2.878$; $\mu_2 = 0.021$; $M_2 = 3.388$; $N_2 = 7.124$; $\mu_3 = 0.251$; $M_3 = 28.926$; $N_3 = 0.241$.

(b) Fit of $S(x)$ obeying GM-CP. SSD $= 115 \cdot 10^{-7}$. Parameters: $\mu_1 = 0.333$; $M_1 = 221.272$; $N_1 = 0.84$; $\mu_2 = 0.028$; $M_2 = 5.483$; $N_2 = 15.192$; $\mu_3 = 0.039$; $M_3 = 14.154$; $N_3 = 42.931$.

(c) Fit of $S(x)$ obeying GS-CP. SSD $= 104 \cdot 10^{-7}$. Parameters: $\mu_1 = 0.045$; $M_1 = 7.431$; $N_1 = 67.219$; $b_1 = 1.283$; $q_1 = 0.034$; $b_2 = 0.32$; $q_2 = 8.223$.

Figure S3. Quadruple 0010-1110. $\epsilon = -0.2010$, $D_{\text{ind}} = 4.512 \cdot 10^{-6}$.

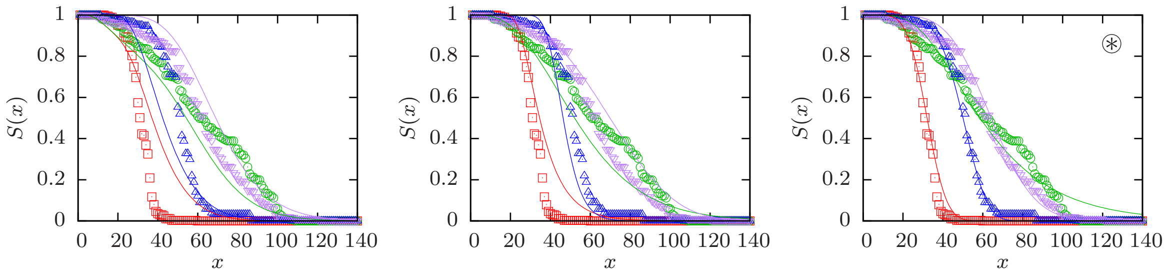

(a) Fit of $S(x)$ obeying CR-CP. SSD $= 2206 \cdot 10^{-7}$. Parameters: $\mu_1 = 0.031$; $M_1 = 12.668$; $N_1 = 2.871$; $\mu_2 = 0.121$; $M_2 = 52.58$; $N_2 = 0.475$; $\mu_3 = 0.276$; $M_3 = 11.285$; $N_3 = 0.037$.

(b) Fit of $S(x)$ obeying GM-CP. SSD $= 787 \cdot 10^{-7}$. Parameters: $\mu_1 = 0.168$; $M_1 = 120.223$; $N_1 = 0.583$; $\mu_2 = 0.022$; $M_2 = 3.715$; $N_2 = 2.6$; $\mu_3 = 0.106$; $M_3 = 173.727$; $N_3 = 2.056$.

(c) Fit of $S(x)$ obeying GS-CP. SSD $= 60 \cdot 10^{-7}$. Parameters: $\mu_1 = 0.027$; $M_1 = 7.571$; $N_1 = 43.458$; $b_1 = 1.699$; $q_1 = 0.018$; $b_2 = 0.364$; $q_2 = 20.918$.

Figure S4. Quadruple 0011-1111. $\epsilon = -0.3786$, $D_{\text{ind}} = 1.8496 \cdot 10^{-6}$.

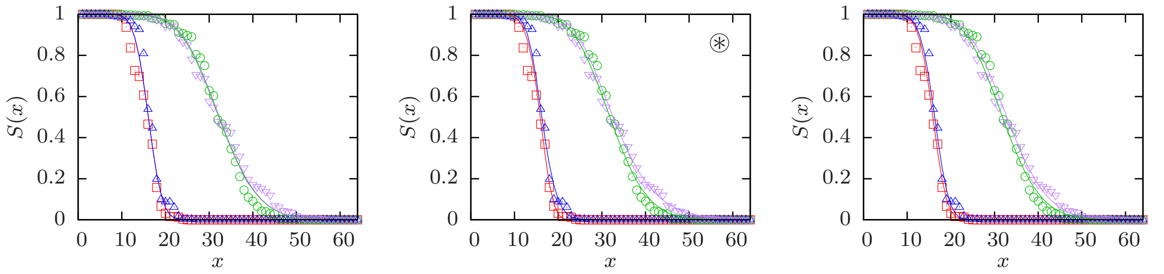

(a) Fit of $S(x)$ obeying CR-CP. SSD $= 2.8 \cdot 10^{-7}$. Parameters: $\mu_1 = 0.062$; $M_1 = 16.136$; $N_1 = 6.701$; $\mu_2 = 0.122$; $M_2 = 22.357$; $N_2 = 20.085$; $\mu_3 = 0.031$; $M_3 = 3.192$; $N_3 = 0.069$.

(b) Fit of $S(x)$ obeying GM-CP. SSD $= 1.8 \cdot 10^{-7}$. Parameters: $\mu_1 = 0.062$; $M_1 = 13.192$; $N_1 = 308.505$; $\mu_2 = 0.062$; $M_2 = 16.377$; $N_2 = 7.44$; $\mu_3 = 0.189$; $M_3 = 43.265$; $N_3 = 5.06$.

(c) Fit of $S(x)$ obeying GS-CP. SSD $= 2.0 \cdot 10^{-7}$. Parameters: $\mu_1 = 0.093$; $M_1 = 16.465$; $N_1 = 49.632$; $b_1 = 0.686$; $q_1 = 0.137$; $b_2 = 0.989$; $q_2 = 0.903$.

Figure S5. Quadruple 0000-1010. $\epsilon = -0.0491$, $D_{\text{ind}} = 5.158 \cdot 10^{-8}$.

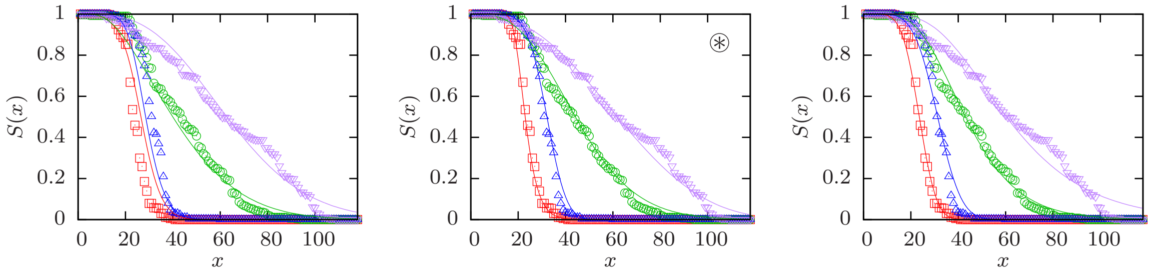

(a) Fit of $S(x)$ obeying CR-CP. SSD $= 123 \cdot 10^{-7}$. Parameters: $\mu_1 = 0.011$; $M_1 = 3.761$; $N_1 = 10.308$; $\mu_2 = 0.102$; $M_2 = 28.854$; $N_2 = 2.835$; $\mu_3 = 0.103$; $M_3 = 6.925$; $N_3 = 0.226$.

(b) Fit of $S(x)$ obeying GM-CP. SSD $= 10 \cdot 10^{-7}$. Parameters: $\mu_1 = 0.234$; $M_1 = 184.357$; $N_1 = 1.27$; $\mu_2 = 0.015$; $M_2 = 3.493$; $N_2 = 8.344$; $\mu_3 = 0.034$; $M_3 = 10.204$; $N_3 = 43.835$.

(c) Fit of $S(x)$ obeying GS-CP. SSD $= 42 \cdot 10^{-7}$. Parameters: $\mu_1 = 0.034$; $M_1 = 7.184$; $N_1 = 52.169$; $b_1 = 1.346$; $q_1 = 0.033$; $b_2 = 0.812$; $q_2 = 0.701$.

Figure S6. Quadruple 0001-1011. $\epsilon = 0.0505$, $D_{\text{ind}} = 1.4136 \cdot 10^{-6}$.

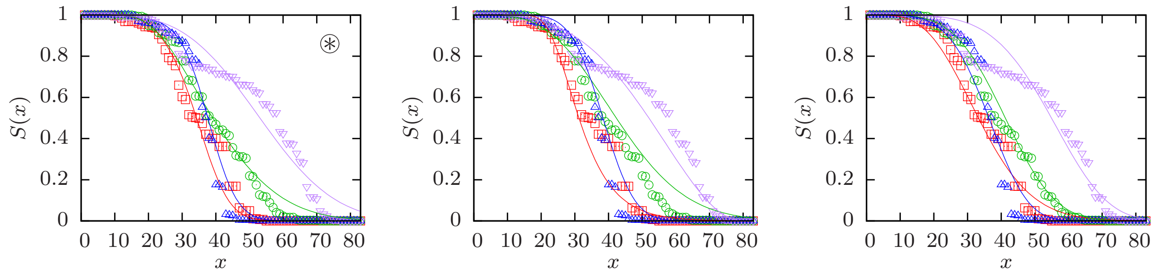

(a) Fit of $S(x)$ obeying CR-CP. SSD $= 74 \cdot 10^{-7}$. Parameters: $\mu_1 = 0.007$; $M_1 = 4.21$; $N_1 = 96.78$; $\mu_2 = 0.065$; $M_2 = 30.902$; $N_2 = 8.206$; $\mu_3 = 0.128$; $M_3 = 28.466$; $N_3 = 0.266$.

(b) Fit of $S(x)$ obeying GM-CP. SSD $= 150 \cdot 10^{-7}$. Parameters: $\mu_1 = 0.156$; $M_1 = 77.711$; $N_1 = 0.857$; $\mu_2 = 0.016$; $M_2 = 4.17$; $N_2 = 13.674$; $\mu_3 = 0.036$; $M_3 = 12.594$; $N_3 = 27.126$.

(c) Fit of $S(x)$ obeying GS-CP. SSD $= 112 \cdot 10^{-7}$. Parameters: $\mu_1 = 0.028$; $M_1 = 4.893$; $N_1 = 7.415$; $b_1 = 0.326$; $q_1 = 25.713$; $b_2 = 0.168$; $q_2 = 762.563$.

Figure S7. Quadruple 0100-1110. $\epsilon = 0.4256$, $D_{\text{ind}} = 5.5274 \cdot 10^{-6}$.

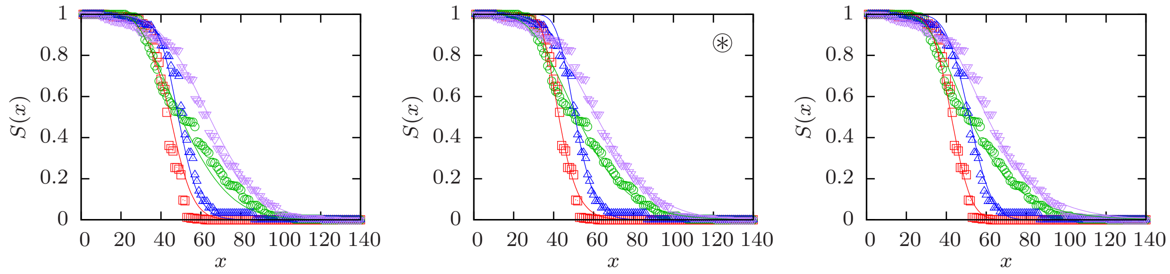

(a) Fit of $S(x)$ obeying CR-CP. SSD $= 43 \cdot 10^{-7}$. Parameters: $\mu_1 = 0.016$; $M_1 = 6.215$; $N_1 = 10.266$; $\mu_2 = 0.104$; $M_2 = 230.211$; $N_2 = 1.756$; $\mu_3 = 0.178$; $M_3 = 200.865$; $N_3 = 0.122$.

(b) Fit of $S(x)$ obeying GM-CP. SSD $= 11 \cdot 10^{-7}$. Parameters: $\mu_1 = 0.118$; $M_1 = 167.359$; $N_1 = 1.668$; $\mu_2 = 0.031$; $M_2 = 6.683$; $N_2 = 2.64$; $\mu_3 = 0.085$; $M_3 = 117.097$; $N_3 = 3.026$.

(c) Fit of $S(x)$ obeying GS-CP. SSD $= 15 \cdot 10^{-7}$. Parameters: $\mu_1 = 0.046$; $M_1 = 19.093$; $N_1 = 13.076$; $b_1 = 1.5$; $q_1 = 0.064$; $b_2 = 0.799$; $q_2 = 1.242$.

Figure S8. Quadruple 0101-1111. $\epsilon = 0.0533$, $D_{\text{ind}} = 8.9449 \cdot 10^{-7}$.

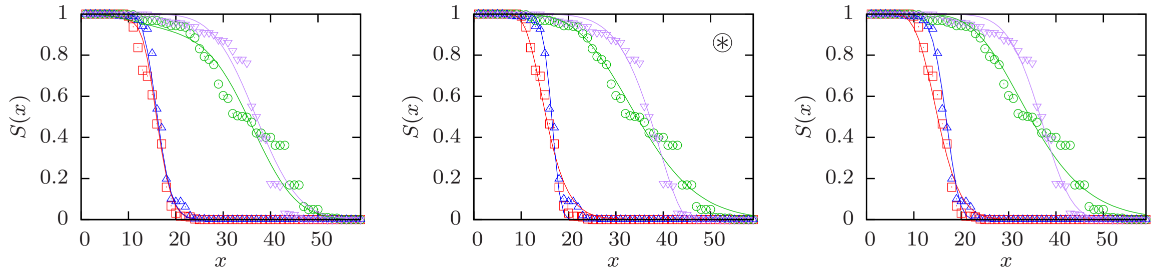

(a) Fit of $S(x)$ obeying CR-CP. SSD $= 21 \cdot 10^{-7}$. Parameters: $\mu_1 = 0.044$; $M_1 = 14.713$; $N_1 = 16.727$; $\mu_2 = 0.234$; $M_2 = 79.603$; $N_2 = 4.034$; $\mu_3 = 0.381$; $M_3 = 8.341$; $N_3 = 0.016$.

(b) Fit of $S(x)$ obeying GM-CP. SSD $= 5.7 \cdot 10^{-7}$. Parameters: $\mu_1 = 0.257$; $M_1 = 47.846$; $N_1 = 1.44$; $\mu_2 = 0.048$; $M_2 = 9.213$; $N_2 = 4.702$; $\mu_3 = 0.17$; $M_3 = 70.315$; $N_3 = 57.407$.

(c) Fit of $S(x)$ obeying GS-CP. SSD $= 7.5 \cdot 10^{-7}$. Parameters: $\mu_1 = 0.151$; $M_1 = 15.776$; $N_1 = 3.546$; $b_1 = 0.483$; $q_1 = 0.639$; $b_2 = 0.464$; $q_2 = 65.955$.

Figure S9. Quadruple 0000-0110. $\epsilon = 0.02915$, $D_{\text{ind}} = 5.2686 \cdot 10^{-7}$.

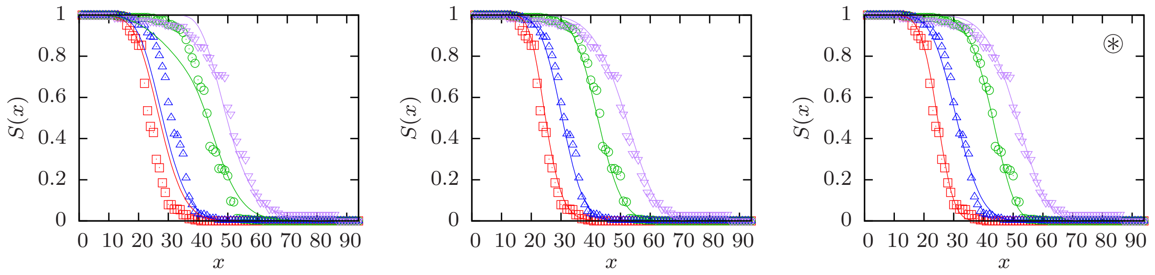

(a) Fit of $S(x)$ obeying CR-CP. SSD $= 182 \cdot 10^{-7}$. Parameters: $\mu_1 = 0.129$; $M_1 = 556.334$; $N_1 = 1.395$; $\mu_2 = 0.078$; $M_2 = 16.077$; $N_2 = 4.439$; $\mu_3 = 0.019$; $M_3 = 4.222$; $N_3 = 5.547$.

(b) Fit of $S(x)$ obeying GM-CP. SSD $= 5.6 \cdot 10^{-7}$. Parameters: $\mu_1 = 0.145$; $M_1 = 57.347$; $N_1 = 3.032$; $\mu_2 = 0.059$; $M_2 = 32.173$; $N_2 = 10.866$; $\mu_3 = 0.058$; $M_3 = 16.361$; $N_3 = 13.96$.

(c) Fit of $S(x)$ obeying GS-CP. SSD $= 3.2 \cdot 10^{-7}$. Parameters: $\mu_1 = 0.052$; $M_1 = 13.054$; $N_1 = 46.497$; $b_1 = 0.402$; $q_1 = 12.705$; $b_2 = 1.01$; $q_2 = 0.245$.

Figure S10. Quadruple 0001-0111. $\epsilon = -0.1090$, $D_{\text{ind}} = 2.0604 \cdot 10^{-7}$.

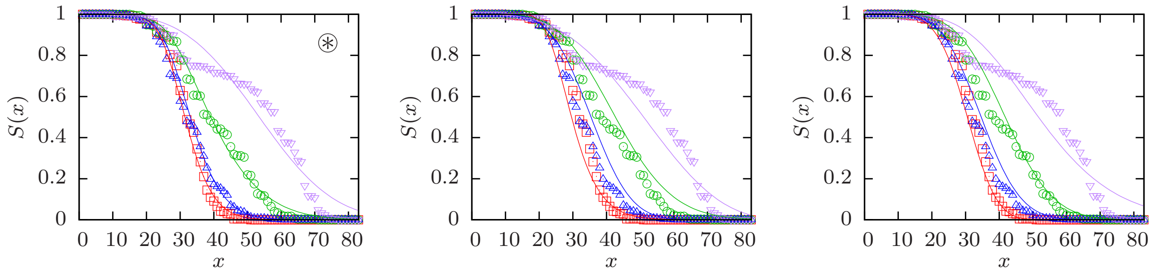

(a) Fit of $S(x)$ obeying CR-CP. SSD $= 42 \cdot 10^{-7}$. Parameters: $\mu_1 = 0.005$; $M_1 = 4.029$; $N_1 = 264.512$; $\mu_2 = 0.105$; $M_2 = 42.38$; $N_2 = 1.849$; $\mu_3 = 0.136$; $M_3 = 81.009$; $N_3 = 0.426$.

(b) Fit of $S(x)$ obeying GM-CP. SSD $= 112 \cdot 10^{-7}$. Parameters: $\mu_1 = 0.136$; $M_1 = 57.683$; $N_1 = 1.555$; $\mu_2 = 0.024$; $M_2 = 6.16$; $N_2 = 10.973$; $\mu_3 = 0.021$; $M_3 = 6.36$; $N_3 = 44.841$.

(c) Fit of $S(x)$ obeying GS-CP. SSD $= 102 \cdot 10^{-7}$. Parameters: $\mu_1 = 0.022$; $M_1 = 7.105$; $N_1 = 122.545$; $b_1 = 1.075$; $q_1 = 0.165$; $b_2 = 1.272$; $q_2 = 0.179$.

Figure S11. Quadruple 1000-1110. $\epsilon = 0.5429$, $D_{\text{ind}} = 4.1708 \cdot 10^{-6}$.

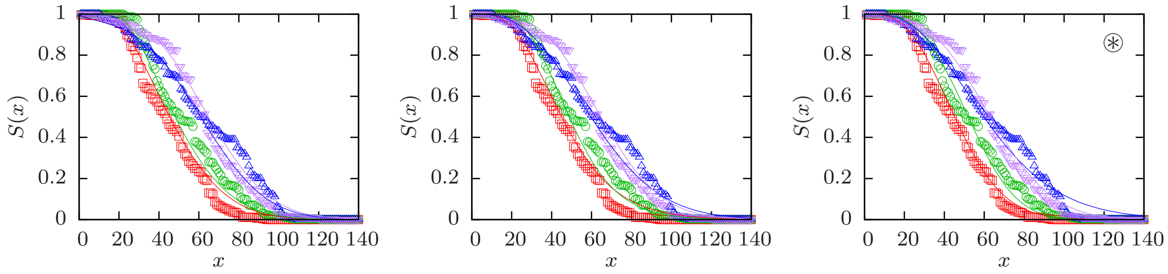

(a) Fit of $S(x)$ obeying CR-CP. SSD $= 49 \cdot 10^{-7}$. Parameters: $\mu_1 = 0.006$; $M_1 = 3.843$; $N_1 = 42.643$; $\mu_2 = 0.037$; $M_2 = 0.528$; $N_2 = 0.043$; $\mu_3 = 0.17$; $M_3 = 99.71$; $N_3 = 0.12$.

(b) Fit of $S(x)$ obeying GM-CP. SSD $= 52 \cdot 10^{-7}$. Parameters: $\mu_1 = 0.032$; $M_1 = 4.671$; $N_1 = 2.037$; $\mu_2 = 0.037$; $M_2 = 7.373$; $N_2 = 2.193$; $\mu_3 = 0.008$; $M_3 = 3.16$; $N_3 = 15.776$.

(c) Fit of $S(x)$ obeying GS-CP. SSD $= 38 \cdot 10^{-7}$. Parameters: $\mu_1 = 0.012$; $M_1 = 3.562$; $N_1 = 14.42$; $b_1 = 0.363$; $q_1 = 13.462$; $b_2 = 1.257$; $q_2 = 0.271$.

Figure S12. Quadruple 1001-1111. $\epsilon = -0.1066$, $D_{\text{ind}} = 2.8763 \cdot 10^{-6}$.

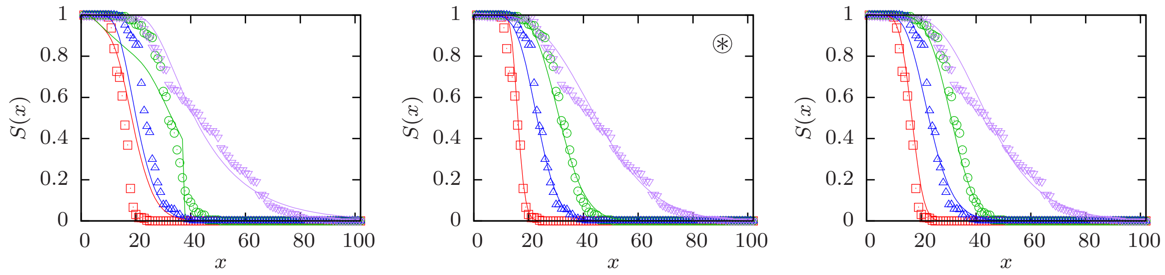

(a) Fit of $S(x)$ obeying CR-CP. SSD $= 480 \cdot 10^{-7}$. Parameters: $\mu_1 = 0.154$; $M_1 = 102.385$; $N_1 = 0.373$; $\mu_2 = 0.147$; $M_2 = 18.818$; $N_2 = 1.495$; $\mu_3 = 0.986$; $M_3 = 27.079$; $N_3 = 0.014$.

(b) Fit of $S(x)$ obeying GM-CP. SSD $= 16 \cdot 10^{-7}$. Parameters: $\mu_1 = 0.294$; $M_1 = 195.589$; $N_1 = 3.594$; $\mu_2 = 0.063$; $M_2 = 14.009$; $N_2 = 5.293$; $\mu_3 = 0.064$; $M_3 = 9.704$; $N_3 = 8.26$.

(c) Fit of $S(x)$ obeying GS-CP. SSD $= 35 \cdot 10^{-7}$. Parameters: $\mu_1 = 0.076$; $M_1 = 10.943$; $N_1 = 26.554$; $b_1 = 0.591$; $q_1 = 0.597$; $b_2 = 1.164$; $q_2 = 0.112$.

Figure S13. Quadruple 0000-1001. $\epsilon = -0.0936$, $D_{\text{ind}} = 2.5148 \cdot 10^{-7}$.

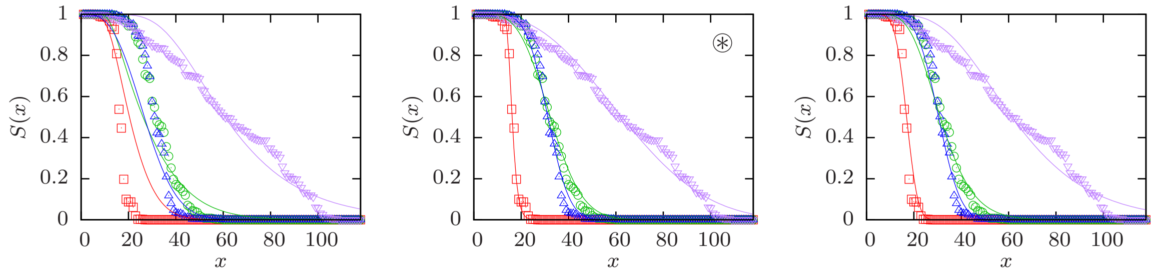

(a) Fit of $S(x)$ obeying CR-CP. SSD $= 1372 \cdot 10^{-7}$. Parameters: $\mu_1 = 0.056$; $M_1 = 15.072$; $N_1 = 0.751$; $\mu_2 = 0.021$; $M_2 = 3.67$; $N_2 = 13.867$; $\mu_3 = 0.303$; $M_3 = 62.549$; $N_3 = 0.179$.

(b) Fit of $S(x)$ obeying GM-CP. SSD $= 12 \cdot 10^{-7}$. Parameters: $\mu_1 = 0.521$; $M_1 = 4295.82$; $N_1 = 1.397$; $\mu_2 = 0.025$; $M_2 = 5.281$; $N_2 = 15.038$; $\mu_3 = 0.037$; $M_3 = 9.214$; $N_3 = 23.377$.

(c) Fit of $S(x)$ obeying GS-CP. SSD $= 54 \cdot 10^{-7}$. Parameters: $\mu_1 = 0.048$; $M_1 = 7.87$; $N_1 = 71.819$; $b_1 = 1.163$; $q_1 = 0.04$; $b_2 = 0.647$; $q_2 = 0.473$.

Figure S14. Quadruple 0010-1011. $\epsilon = 0.0013$, $D_{\text{ind}} = 1.1353 \cdot 10^{-6}$.

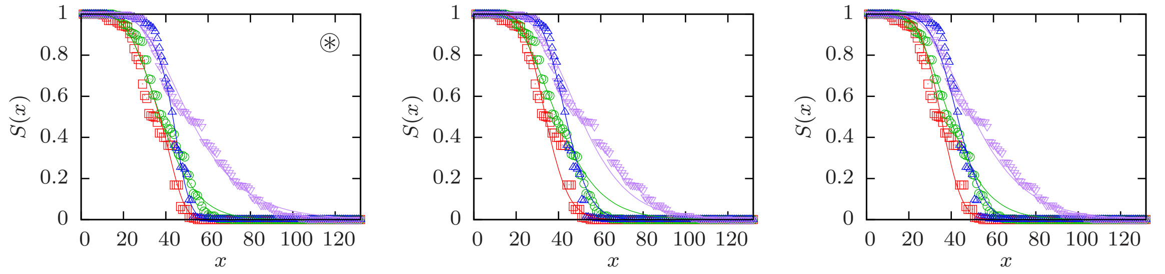

(a) Fit of $S(x)$ obeying CR-CP. SSD $= 14 \cdot 10^{-7}$. Parameters: $\mu_1 = 0.056$; $M_1 = 12.074$; $N_1 = 1.015$; $\mu_2 = 0.085$; $M_2 = 132.234$; $N_2 = 8.756$; $\mu_3 = 0.047$; $M_3 = 6.797$; $N_3 = 1.366$.

(b) Fit of $S(x)$ obeying GM-CP. SSD $= 36 \cdot 10^{-7}$. Parameters: $\mu_1 = 0.061$; $M_1 = 14.367$; $N_1 = 4.576$; $\mu_2 = 0.05$; $M_2 = 7.445$; $N_2 = 1.796$; $\mu_3 = 0.062$; $M_3 = 26.979$; $N_3 = 4.224$.

(c) Fit of $S(x)$ obeying GS-CP. SSD $= 37 \cdot 10^{-7}$. Parameters: $\mu_1 = 0.031$; $M_1 = 9.327$; $N_1 = 33.307$; $b_1 = 1.741$; $q_1 = 0.06$; $b_2 = 0.885$; $q_2 = 0.674$.

Figure S15. Quadruple 0100-1101. $\epsilon = 0.0541$, $D_{\text{ind}} = 7.928 \cdot 10^{-7}$.

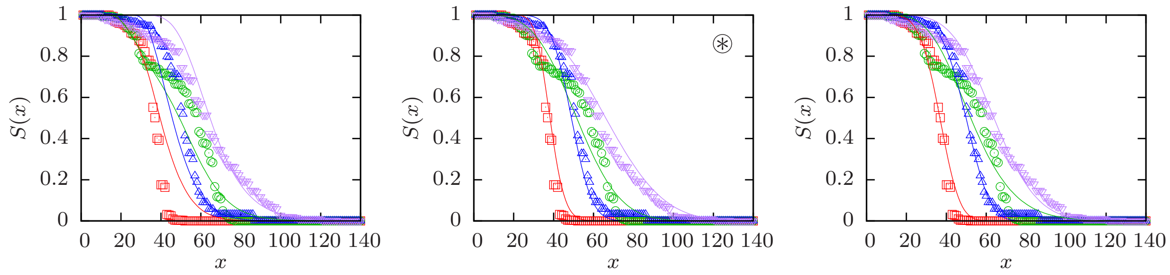

(a) Fit of $S(x)$ obeying CR-CP. SSD $= 563 \cdot 10^{-7}$. Parameters: $\mu_1 = 0.066$; $M_1 = 65.436$; $N_1 = 1.403$; $\mu_2 = 0.175$; $M_2 = 648.33$; $N_2 = 0.395$; $\mu_3 = 0.244$; $M_3 = 163.88$; $N_3 = 0.085$.

(b) Fit of $S(x)$ obeying GM-CP. SSD $= 96 \cdot 10^{-7}$. Parameters: $\mu_1 = 0.082$; $M_1 = 49.242$; $N_1 = 6.162$; $\mu_2 = 0.01$; $M_2 = 4.529$; $N_2 = 39.916$; $\mu_3 = 0.041$; $M_3 = 21.701$; $N_3 = 16.107$.

(c) Fit of $S(x)$ obeying GS-CP. SSD $= 102 \cdot 10^{-7}$. Parameters: $\mu_1 = 0.022$; $M_1 = 8.16$; $N_1 = 92.495$; $b_1 = 1.485$; $q_1 = 0.037$; $b_2 = 0.581$; $q_2 = 3.808$.

Figure S16. Quadruple 0110-1111. $\epsilon = -0.2212$, $D_{\text{ind}} = 5.498 \cdot 10^{-6}$.

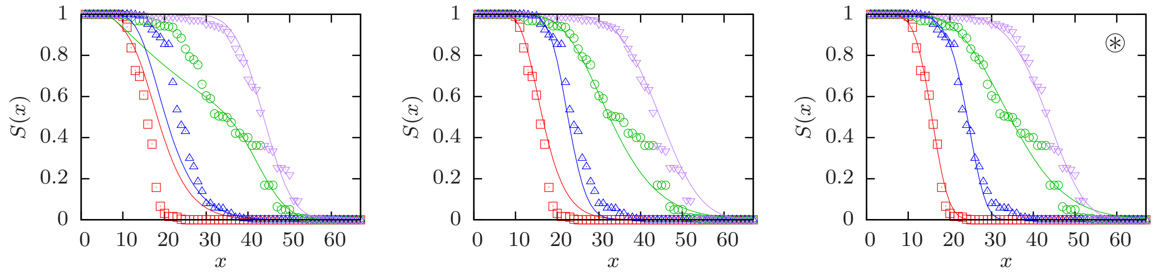

(a) Fit of $S(x)$ obeying CR-CP. SSD $= 375 \cdot 10^{-7}$. Parameters: $\mu_1 = 0.061$; $M_1 = 45.843$; $N_1 = 17.289$; $\mu_2 = 0.175$; $M_2 = 27.55$; $N_2 = 1.162$; $\mu_3 = 0.623$; $M_3 = 91.305$; $N_3 = 0.036$.

(b) Fit of $S(x)$ obeying GM-CP. SSD $= 40 \cdot 10^{-7}$. Parameters: $\mu_1 = 0.214$; $M_1 = 31.038$; $N_1 = 1.403$; $\mu_2 = 0.08$; $M_2 = 16.463$; $N_2 = 2.111$; $\mu_3 = 0.135$; $M_3 = 45.902$; $N_3 = 6.025$.

(c) Fit of $S(x)$ obeying GS-CP. SSD $= 4.9 \cdot 10^{-7}$. Parameters: $\mu_1 = 0.063$; $M_1 = 9.545$; $N_1 = 57.884$; $b_1 = 0.809$; $q_1 = 0.067$; $b_2 = 0.288$; $q_2 = 231.924$.

Figure S17. Quadruple 0000-0101. $\epsilon = -0.1737$, $D_{\text{ind}} = 3.821 \cdot 10^{-7}$.

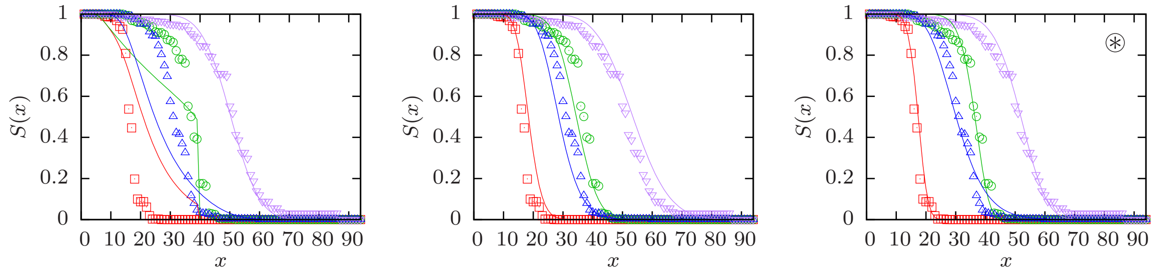

(a) Fit of $S(x)$ obeying CR-CP. SSD $= 1921 \cdot 10^{-7}$. Parameters: $\mu_1 = 0.04$; $M_1 = 23.959$; $N_1 = 21.942$; $\mu_2 = 0.207$; $M_2 = 35.102$; $N_2 = 0.415$; $\mu_3 = 0.936$; $M_3 = 241.971$; $N_3 = 0.021$.

(b) Fit of $S(x)$ obeying GM-CP. SSD $= 140 \cdot 10^{-7}$. Parameters: $\mu_1 = 0.15$; $M_1 = 30.119$; $N_1 = 4.654$; $\mu_2 = 0.054$; $M_2 = 19.871$; $N_2 = 18.907$; $\mu_3 = 0.104$; $M_3 = 31.716$; $N_3 = 3.317$.

(c) Fit of $S(x)$ obeying GS-CP. SSD $= 21 \cdot 10^{-7}$. Parameters: $\mu_1 = 0.09$; $M_1 = 18.485$; $N_1 = 51.204$; $b_1 = 0.309$; $q_1 = 53.435$; $b_2 = 0.966$; $q_2 = 0.047$.

Figure S18. Quadruple 0010-0111. $\epsilon = -0.2846$, $D_{\text{ind}} = 3.211 \cdot 10^{-7}$.

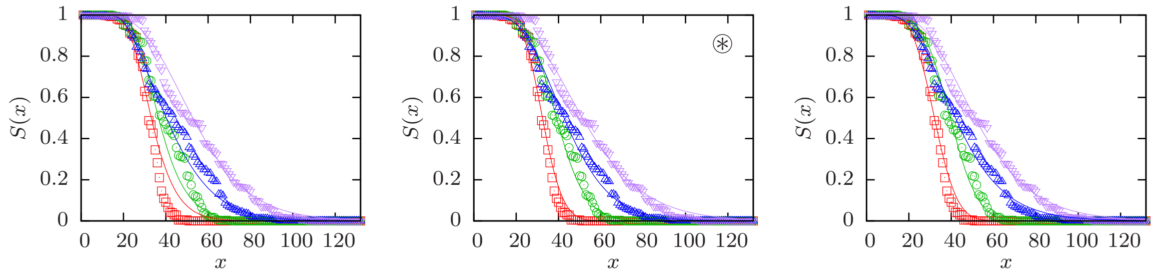

(a) Fit of $S(x)$ obeying CR-CP. SSD $= 47 \cdot 10^{-7}$. Parameters: $\mu_1 = 0.036$; $M_1 = 7.317$; $N_1 = 1.935$; $\mu_2 = 0.239$; $M_2 = 193.206$; $N_2 = 0.092$; $\mu_3 = 0.143$; $M_3 = 97.319$; $N_3 = 0.537$.

(b) Fit of $S(x)$ obeying GM-CP. SSD $= 9.2 \cdot 10^{-7}$. Parameters: $\mu_1 = 0.051$; $M_1 = 12.281$; $N_1 = 9.083$; $\mu_2 = 0.043$; $M_2 = 10.075$; $N_2 = 5.283$; $\mu_3 = 0.034$; $M_3 = 5.975$; $N_3 = 2.96$.

(c) Fit of $S(x)$ obeying GS-CP. SSD $= 12 \cdot 10^{-7}$. Parameters: $\mu_1 = 0.034$; $M_1 = 7.795$; $N_1 = 17.329$; $b_1 = 0.795$; $q_1 = 1.089$; $b_2 = 1.569$; $q_2 = 0.077$.

Figure S19. Quadruple 1000-1101. $\epsilon = -0.0390$, $D_{\text{ind}} = 5.6519 \cdot 10^{-7}$.

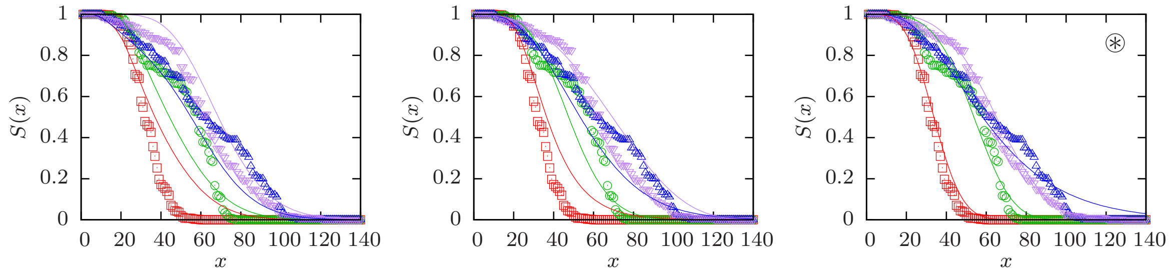

(a) Fit of $S(x)$ obeying CR-CP. SSD $= 1431 \cdot 10^{-7}$. Parameters: $\mu_1 = 0.025$; $M_1 = 9.821$; $N_1 = 4.574$; $\mu_2 = 0.217$; $M_2 = 9.762$; $N_2 = 0.044$; $\mu_3 = 0.212$; $M_3 = 124.707$; $N_3 = 0.133$.

(b) Fit of $S(x)$ obeying GM-CP. SSD $= 896 \cdot 10^{-7}$. Parameters: $\mu_1 = 0.084$; $M_1 = 13.134$; $N_1 = 0.923$; $\mu_2 = 0.017$; $M_2 = 5.175$; $N_2 = 14.124$; $\mu_3 = 0.015$; $M_3 = 3.588$; $N_3 = 5.217$.

(c) Fit of $S(x)$ obeying GS-CP. SSD $= 91 \cdot 10^{-7}$. Parameters: $\mu_1 = 0.012$; $M_1 = 4.263$; $N_1 = 79.243$; $b_1 = 0.17$; $q_1 = 143.301$; $b_2 = 2.322$; $q_2 = 0.018$.

Figure S20. Quadruple 1010-1111. $\epsilon = -0.4436$, $D_{\text{ind}} = 8.582 \cdot 10^{-6}$.

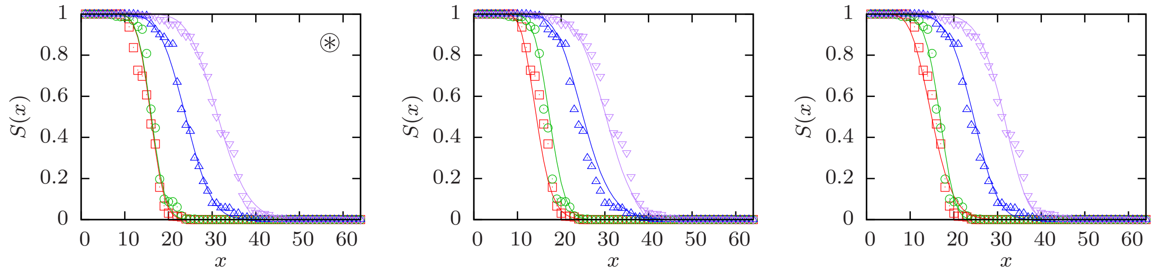

(a) Fit of $S(x)$ obeying CR-CP. SSD $= 1.9 \cdot 10^{-7}$. Parameters: $\mu_1 = 0.087$; $M_1 = 32.73$; $N_1 = 6.075$; $\mu_2 = 0.089$; $M_2 = 17.311$; $N_2 = 5.206$; $\mu_3 = 0.146$; $M_3 = 28.377$; $N_3 = 11.54$.

(b) Fit of $S(x)$ obeying GM-CP. SSD $= 13 \cdot 10^{-7}$. Parameters: $\mu_1 = 0.219$; $M_1 = 36.637$; $N_1 = 3.13$; $\mu_2 = 0.134$; $M_2 = 26.819$; $N_2 = 10.507$; $\mu_3 = 0.114$; $M_3 = 29.223$; $N_3 = 3.642$.

(c) Fit of $S(x)$ obeying GS-CP. SSD $= 3.3 \cdot 10^{-7}$. Parameters: $\mu_1 = 0.127$; $M_1 = 12.269$; $N_1 = 4.812$; $b_1 = 0.472$; $q_1 = 38.091$; $b_2 = 0.399$; $q_2 = 9.404$.

Figure S21. Quadruple 0000-0011. $\epsilon = 0.2791$, $D_{\text{ind}} = 5.504 \cdot 10^{-8}$.

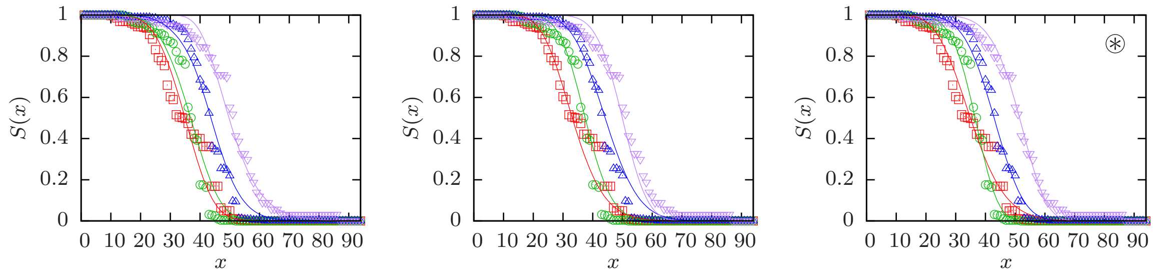

(a) Fit of $S(x)$ obeying CR-CP. SSD $= 43 \cdot 10^{-7}$. Parameters: $\mu_1 = 0.109$; $M_1 = 230.92$; $N_1 = 1.536$; $\mu_2 = 0.044$; $M_2 = 11.606$; $N_2 = 2.725$; $\mu_3 = 0.038$; $M_3 = 10.119$; $N_3 = 11.36$.

(b) Fit of $S(x)$ obeying GM-CP. SSD $= 46 \cdot 10^{-7}$. Parameters: $\mu_1 = 0.066$; $M_1 = 14.96$; $N_1 = 3.886$; $\mu_2 = 0.06$; $M_2 = 22.372$; $N_2 = 7.929$; $\mu_3 = 0.048$; $M_3 = 18.999$; $N_3 = 7.437$.

(c) Fit of $S(x)$ obeying GS-CP. SSD $= 12 \cdot 10^{-7}$. Parameters: $\mu_1 = 0.052$; $M_1 = 11.486$; $N_1 = 4.918$; $b_1 = 0.504$; $q_1 = 36.417$; $b_2 = 0.509$; $q_2 = 11.903$.

Figure S22. Quadruple 0100-0111. $\epsilon = 0.1075$, $D_{\text{ind}} = 7.274 \cdot 10^{-7}$.

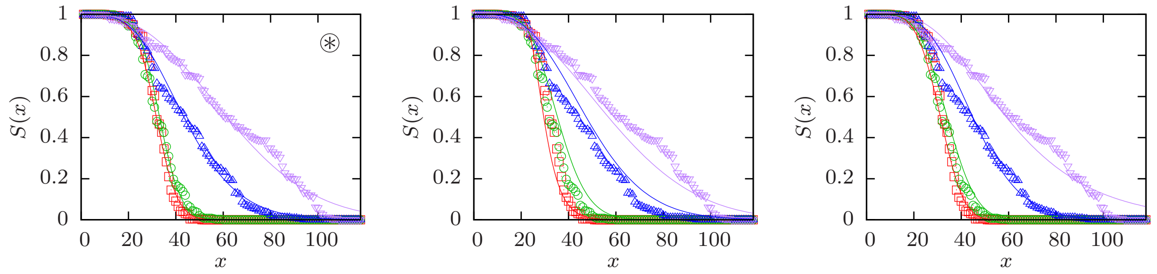

(a) Fit of $S(x)$ obeying CR-CP. SSD $= 13 \cdot 10^{-7}$. Parameters: $\mu_1 = 0.004$; $M_1 = 2.796$; $N_1 = 37.575$; $\mu_2 = 0.049$; $M_2 = 8.334$; $N_2 = 0.922$; $\mu_3 = 0.076$; $M_3 = 26.672$; $N_3 = 4.727$.

(b) Fit of $S(x)$ obeying GM-CP. SSD $= 125 \cdot 10^{-7}$. Parameters: $\mu_1 = 0.189$; $M_1 = 211.854$; $N_1 = 1.168$; $\mu_2 = 0.047$; $M_2 = 9.778$; $N_2 = 5.316$; $\mu_3 = 0.018$; $M_3 = 4.046$; $N_3 = 6.617$.

(c) Fit of $S(x)$ obeying GS-CP. SSD $= 52 \cdot 10^{-7}$. Parameters: $\mu_1 = 0.018$; $M_1 = 6.104$; $N_1 = 124.647$; $b_1 = 1.29$; $q_1 = 0.227$; $b_2 = 1.599$; $q_2 = 0.038$.

Figure S23. Quadruple 1000-1011. $\epsilon = 0.4131$, $D_{\text{ind}} = 1.558 \cdot 10^{-6}$.

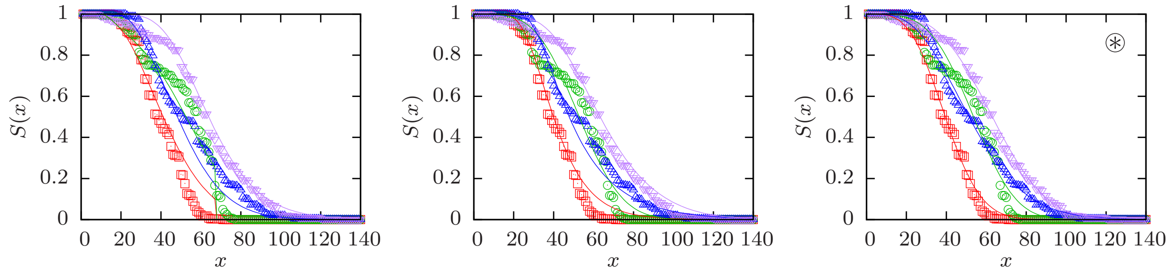

(a) Fit of $S(x)$ obeying CR-CP. SSD $= 82 \cdot 10^{-7}$. Parameters: $\mu_1 = 0.04$; $M_1 = 14.49$; $N_1 = 1.93$; $\mu_2 = 0.166$; $M_2 = 91.663$; $N_2 = 0.14$; $\mu_3 = 0.552$; $M_3 = 935.934$; $N_3 = 0.018$.

(b) Fit of $S(x)$ obeying GM-CP. SSD $= 68 \cdot 10^{-7}$. Parameters: $\mu_1 = 0.085$; $M_1 = 19.131$; $N_1 = 0.96$; $\mu_2 = 0.008$; $M_2 = 4.462$; $N_2 = 73.286$; $\mu_3 = 0.136$; $M_3 = 104.688$; $N_3 = 0.362$.

(c) Fit of $S(x)$ obeying GS-CP. SSD $= 44 \cdot 10^{-7}$. Parameters: $\mu_1 = 0.012$; $M_1 = 4.102$; $N_1 = 36.583$; $b_1 = 0.331$; $q_1 = 16.172$; $b_2 = 1.432$; $q_2 = 0.157$.

Figure S24. Quadruple 1100-1111. $\epsilon = -0.1818$, $D_{\text{ind}} = 5.075 \cdot 10^{-6}$.
